# Efficacy and safety of semaglutide on weight loss in obese or overweight patients without diabetes: A systematic review and meta-analysis of randomized controlled trials

**DOI:** 10.3389/fphar.2022.935823

**Published:** 2022-09-14

**Authors:** Xueqin Gao, Xiaoli Hua, Xu Wang, Wanbin Xu, Yu Zhang, Chen Shi, Ming Gu

**Affiliations:** ^1^ Department of Pharmacy, Union Hospital, Tongji Medical College, Huazhong University of Science and Technology, Wuhan, China; ^2^ Hubei Province Clinical Research Center for Precision Medicine for Critical Illness, Wuhan, China

**Keywords:** weight loss, semaglutide, obesity, without diabetes, meta-analysis

## Abstract

**Objectives:** This study aims to explore the weight loss effect and safety of semaglutide as a conventional anti-obesity drug systematically in obese or overweight patients without diabetes.

**Methods:** The randomized controlled trials (RCTs) of semaglutide in obese or overweight patients without diabetes were retrieved from PubMed, Cochrane Library, EMBASE, and ClinicalTrials.gov from database inception until 2 May 2022. Data extraction and quality assessment of studies meeting the inclusion criteria were performed, and statistical analysis was conducted by Review Manager 5.3 and Stata 14.

**Results:** Eight studies involving 4,567 patients were enrolled in the meta-analysis. Compared with placebo, semaglutide induced a significant body weight loss (MD: −10.09%; 95% CI: −11.84 to −8.33; *p* ˂ 0.00001), elicited a larger reduction in body mass index (MD: −3.71 kg/m^2^; 95% CI: −4.33 to −3.09; *p* ˂ 0.00001) and waist circumference (MD: −8.28 cm; 95% CI: −9.51 to −7.04; *p* ˂ 0.00001), achieved weight loss of more than 5, 10, 15, and 20% with a higher proportion of participants. Semaglutide exhibited a positive effect on blood pressure, C-reactive protein, and lipid profiles, expressed more adverse effects than placebo, mainly gastrointestinal reactions. The results were stable and reliable with dose-dependence.

**Conclusion:** Semaglutide indicated a significant weight loss with an acceptable safety for obese or overweight patients without diabetes.

## 1 Introduction

Obesity (BMI ≥ 30 kg/m^2^) and overweight (BMI ≥ 27 kg/m^2^), characterized as chronic diseases and major public health issues (Ng et al., 2014; Milano et al., 2020), are associated with an increased risk of diabetes, hypertension, hyperlipidemia, stroke, and malignant tumor (Guh et al., 2009). Sustained clinically meaningful weight loss is a major goal in preventing the progression of diabetes and other obesity-related complications (Sharma and Garber, 2009). It is generally known that diet and exercise intervention are the most effective ways to lose weight, but long-term adherence is challenging (Dombrowski et al., 2014; Webb and Wadden, 2017). The safety and tolerability of anti-obesity drugs are unsatisfactory (Yanovski and Yanovski, 2014; Bessesen and Van Gaal, 2018), consequently limiting clinical use, while the safety concerns and cost of bariatric surgery also obstruct their application (Arterburn et al., 2020).

Liraglutide (Saxenda^®^, 3 mg), a glucagon-like peptide 1 receptor agonist (GLP-1RA) used for the treatment of type 2 diabetes, was approved by the FDA in 2014 for weight management in obese or overweight adults without diabetes (Pi-Sunyer et al., 2015; Scott 2015). Since then, GLP-1RA has created a new field for obesity treatment (Zhang et al., 2015; Christensen et al., 2019; Ryan et al., 2021). By activating the GLP-1 receptor, GLP-1RA enhances insulin secretion and inhibits glucagon secretion to lower blood glucose, ingeniously suppressing appetite, increasing satiety, and delaying gastric emptying, thus achieving weight reduction (Torekov et al., 2011; Monami et al., 2012; Lee 2021). Compared with once-daily liraglutide, once-weekly subcutaneous semaglutide, a novel longer-acting GLP-1RA, was approved by the FDA for weight management in the United States on 4 June 2021 (Lau et al., 2015; Hall et al., 2018; Kushner et al., 2020; Singh et al., 2022).

Meta-analyses evaluating the efficacy and safety between semaglutide and placebo in obese patients have been conducted previously. Vosoughi et al. (2021) and He et al. (2022) both explored the weight loss effect of once-weekly semaglutide for obesity, involving patients with or without diabetes. Iqbal et al. (2022) and Zhong et al. (2022) designed the study in non-diabetic patients intriguingly, but only contained three or four RCTs about semaglutide and placebo. Additionally, all the aforementioned studies focused on 2.4 mg-dosed semaglutide and placebo. In order to explore the role of semaglutide as a conventional weight-lowering drug for obese patients without diabetes, as well as the relationship between the dose of semaglutide and efficacy, and the other potentially beneficial effects, a latest and comprehensive meta-analysis based on different doses was carried out to assess the weight loss effect between semaglutide and placebo in obese or overweight patients without diabetes, further providing more favorable strategies for clinical individualized medication with obesity.

## 2 Materials and methods

### 2.1 Study selection criteria

The inclusion criteria were listed as follows: 1) the study design must be randomized controlled trials (RCTs); 2) the participants were obese or overweight patients over 18 years of age without diabetes; 3) the intervention agent was semaglutide, and placebo was comparison one; 4) the outcome measures may include weight-related indicators and safety. Moreover, we excluded some studies, including duplicated, non-RCT, reviews, and those in which participants were diagnosed with type 1 or type 2 diabetes mellitus.

### 2.2 Literature retrieval

Literature searches were performed in PubMed, Cochrane Library, EMBASE, and ClinicalTrials.gov from their inception until 2 May 2022, using the search terms “obesity,” “semaglutide,” and “randomized controlled trial.” The retrieval was limited to English-language articles. The specific search strategies are referred to in Supplementary Table S1 in the Supplementary Material.

### 2.3 Data extraction

Two authors extracted data, respectively, according to the designed data extraction table. They not only extracted the original data, but also processed or converted them uniformly, such as converting standard error or confidence interval into standard deviation, and differences within and between groups should be taken into account as well. In case of disagreement during data extraction, a third researcher will intervene for arbitration.

The extracted data consisted of research information, characteristics of patients, outcome measures, and safety. The details were as follows: The research information demonstrated the study ID (the first author’s last name and publication year), NCT number, intervention and control drugs, and duration. The characteristics of patients contained the total number, gender, age, body weight (BW), body mass index (BMI), waist circumference (WC), and proportions with or without comorbidities at baseline. The measures related to efficacy outcome were recorded, including the mean changes in 1) weight-related indicators (BW, BMI, WC, and the proportion of participants who achieved weight loss of more than 5, 10, 15, and 20%), 2) cardiovascular-related indicators [systolic blood pressure (SBP), diastolic blood pressure (DBP), and C-reactive protein (CRP)] and 3) the lipid-related indicators [total cholesterol (TC), high-density lipoprotein cholesterol (HDL), low-density lipoprotein cholesterol (LDL), very low density lipoprotein cholesterol (VLDL), triglycerides (TG), and free fatty acids (FFAs)]. The safety outcomes considered the proportion of participants with adverse events (AEs), serious adverse events (SAEs), adverse events leading to discontinuation (DAEs), and specific side effects, such as hypoglycemia, nausea, and diarrhea.

### 2.4 Quality assessment

The methodological quality of the included RCTs was assessed separately by two investigators through the Cochrane collaboration’s tool for assessing risk of bias, involving the following items: 1) random sequence generation, 2) allocation concealment, 3) blinding of participants and personnel, 4) blinding of outcome assessment, 5) incomplete outcome data, 6) selective reporting, and 7) other bias. The items were judged as “low risk,” “unclear risk,” and “high risk” for each study, and a third researcher intervened to settle disputes through negotiation.

### 2.5 Statistical analysis

All analyses were conducted by Review Manager 5.3 and Stata 14, which are produced by Cochrane Collaboration. When merging statistics, continuous data were performed as the mean difference (MD) to express effect size, while dichotomous data were adopted as the risk ratio (RR), and 95% confidence intervals were used for interval estimation correspondingly. Tests for heterogeneity were estimated using the Q test and evaluated by the Ⅰ^2^ test. The fixed effect model was used when there was no statistical heterogeneity in the included studies (Ⅰ2 ˂ 50% and *p* ˃ 0.10); otherwise, the random effect model was represented (Ⅰ^2^ ≥ 50% or *p* ˂ 0.10) (Chen and Benedetti, 2017). The meta-analysis results were shown by forest plots, and the publication biases were observed by funnel plots (Biljana et al., 1999). Moreover, the sensitivity analysis was assessed to evaluate the stability and reliability of the results. Subgroup and meta-regression analyses were elaborated to explore the heterogeneities in different baseline variables.

## 3 Results

### 3.1 Retrieval results

**FIGURE 1:**
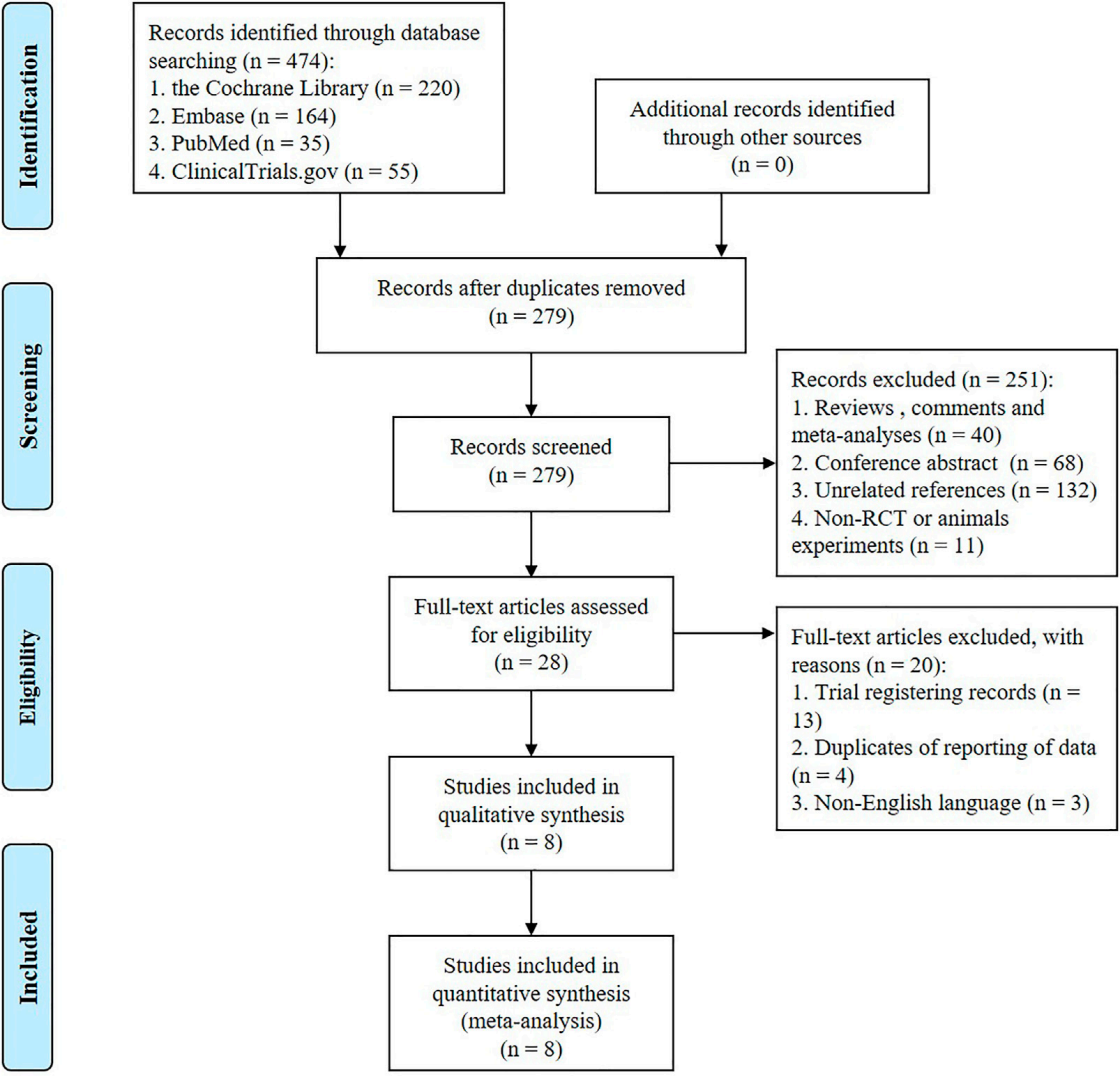
Flow diagram of literature retrieval.

A total of 474 studies were preliminarily retrieved from the databases, among which 55 were from ClinicalTrials. A total 279 were obtained after eliminating duplicates. After screening the titles and abstracts, 251 were excluded based on the aforementioned inclusion and exclusion criteria. The remaining 28 studies were reviewed in full text, excluding trial registration records, duplicates of reporting, and non-English literature. Subsequently, eight studies involving 4,567 participants (Wilding et al., 2021; Wadden et al., 2021; Rubino et al., 2021; Rubino et al., 2022; O'Neil et al., 2018; Friedrichsen et al., 2021; Hjerpsted et al., 2018; Jensterle et al., 2021) were included in the meta-analysis. The flow diagram of literature retrieval is displayed in Figure 1.

### 3.2 Study characteristics

The baseline characteristics of included studies are summarized in Table 1. All eight studies were conducted on non-diabetic obese patients with an average baseline BMI of 33.8–40.1 kg/m^2^, and the individuals were all adults over 18 years of age (Wilding et al., 2021; Wadden et al., 2021; Rubino et al., 2021; Rubino et al., 2022; O'Neil et al., 2018; Friedrichsen et al., 2021; Hjerpsted et al., 2018; Jensterle et al., 2021). Only one study included obese women with polycystic ovary syndrome (Jensterle et al., 2021). Semaglutide was administered subcutaneously in the included eight studies, five of which were 2.4 mg/week (Friedrichsen et al., 2021; Rubino et al., 2021; Wadden et al., 2021; Wilding et al., 2021; Rubino et al., 2022), two were 1.0 mg/week (Hjerpsted et al., 2018; Jensterle et al., 2021), and one was 0.05, 0.1, 0.2, 0.3, or 0.4 mg/day with dose escalation every fourth week, as well as every second week in the fast dose escalation group (FE) (O'Neil et al., 2018). Placebo was the comparator in all studies, two of which included liraglutide (Rubino et al., 2022; O'Neil et al., 2018). The follow-up durations ranged from 12 to 68 weeks. A total of seven studies mentioned body weight changes (Wilding et al., 2021; Wadden et al., 2021; Rubino et al., 2021; Rubino et al., 2022; O'Neil et al., 2018; Friedrichsen et al., 2021; Jensterle et al., 2021), while six reported changes in WC (Wilding et al., 2021; Wadden et al., 2021; Rubino et al., 2021; Rubino et al., 2022; O'Neil et al., 2018; Jensterle et al., 2021), five displayed the proportion of participants who achieved weight loss of more than 5, 10, 15, and 20% (Wilding et al., 2021; Wadden et al., 2021; Rubino et al., 2021; Rubino et al., 2022; O'Neil et al., 2018), five recorded changes of BMI, blood pressure, CRP, and lipid profiles (Wilding et al., 2021; Wadden et al., 2021; Rubino et al., 2021; Rubino et al., 2022; O'Neil et al., 2018), and all studies provided the adverse events.

**TABLE 1:** Characteristics of included studies.

	Study ID	NCT number	Intervention and comparison	Patient	Female, n (%)	Age, year	BW (kg)	BMI (kg/m^2^)	WC (cm)	Study duration (week)
1	Wilding et al. (2021)	NCT03548935	Semaglutide 2.4 mg QW	1,306	955 (73.1)	46 ± 13	105.4 ± 22.1	37.8 ± 6.7	114.6 ± 14.8	68
			Placebo	655	498 (76.0)	47 ± 12	105.2 ± 21.5	38.0 ± 6.5	114.8 ± 14.4	
2	Wadden et al. (2021)	NCT03611582	Semaglutide 2.4 mg QW	407	315 (77.4)	46 ± 13	106.9 ± 22.8	38.1 ± 6.7	113.6 ± 15.1	68
			Placebo	204	180 (88.2)	46 ± 13	103.7 ± 22.9	37.8 ± 6.9	111.8 ± 16.2	
3	Rubino et al. (2021)	NCT03548987	Semaglutide 2.4 mg QW	535	429 (80.2)	47 ± 12	96.5 ± 22.5	34.5 ± 6.9	105.5 ± 15.9	68
			Placebo	268	205 (76.5)	46 ± 12	95.4 ± 22.7	34.1 ± 7.1	104.7 ± 16.9	
4	Rubino et al. (2022)	NCT04074161	Semaglutide 2.4 mg QW	126	102 (81.0)	48 ± 14	102.5 ± 25.3	37.0 ± 7.4	111.8 ± 16.3	68
			Placebo	85	66 (77.6)	51 ± 12	108.8 ± 23.1	38.8 ± 6.5	115.4 ± 15.1	
5	O'Neil et al. (2018)	NCT02453711	Semaglutide 0.05 mg QD	103	67 (65.0)	47 ± 13	111.3 ± 23.2	39.1 ± 6.5	117.0 ± 14.6	52
			Semaglutide 0.1 mg QD	102	66 (65.0)	45 ± 13	111.3 ± 21.5	39.6 ± 7.4	117.1 ± 13.7	
			Semaglutide 0.2 mg QD	103	66 (64.0)	44 ± 11	114.5 ± 24.5	40.1 ± 6.9	119.1 ± 15.2	
			Semaglutide 0.3 mg QD	103	66 (64.0)	47 ± 12	111.5 ± 23.0	39.6 ± 7.1	118.1 ± 15.1	
			Semaglutide 0.4 mg QD	102	66 (65.0)	48 ± 13	113.2 ± 26.4	39.9 ± 8.8	119.0 ± 16.3	
			Semaglutide 0.3 mg FE QD	102	66 (65.0)	47 ± 12	108.1 ± 22.1	38.2 ± 6.5	117.1 ± 13.8	
			Semaglutide 0.4 mg FE QD	103	67 (65.0)	46 ± 14	109.6 ± 21.3	38.5 ± 5.9	116.8 ± 15.5	
			Placebo	136	88 (65.0)	46 ± 13	114.2 ± 25.4	40.1 ± 7.2	119.5 ± 15.9	
6	Friedrichsen et al. (2021)	NCT03842202	Semaglutide 2.4 mg QW	36	12 (33.3)	40.7 ± 12.2	106.2 ± 16.2	34.2 ± 3.0	NA	20
			Placebo	36	16 (44.4)	45.0 ± 9.5	104.9 ± 14.0	34.6 ± 3.1	NA	
7	Hjerpsted et al. (2018)	NCT02079870	Semaglutide 1.0 mg QW	15	5 (33.3)	42 ± 11	101.3 ± 10.5	33.8 ± 2.5	NA	12
			Placebo	15	5 (33.3)	42 ± 11	101.3 ± 10.5	33.8 ± 2.5	NA	
8	Jensterle et al. (2021)	NCT04263415	Semaglutide 1.0 mg QW	13	13 (100)	33.7 ± 5.3	100.8 ± 11.8	36.8 ± 3.9	106.3 ± 10.1	16
			Placebo	12	12 (100)	33.7 ± 5.3	98.8 ± 13.1	35.4 ± 3.8	107.1 ± 9.9	

The BMI and proportions of individuals with or without comorbidities are collected in Supplementary Table S2. Four studies enrolled adults with BMI of 30 or greater or a BMI of 27 or greater with at least 1 weight-related comorbidity, and the average proportion of individuals without comorbidities was 24.4% (Rubino et al., 2021; Wadden et al., 2021; Wilding et al., 2021; Rubino et al., 2022). The other four studies contained adults with a BMI of 30 or greater without comorbidities totally (O'Neil et al., 2018; Friedrichsen et al., 2021; Hjerpsted et al., 2018; Jensterle et al., 2021). The proportions of individuals with different types of comorbidities at baseline were presented, especially the metabolic syndrome, such as dyslipidemia, hypertension, obstructive sleep apnea, and cardiovascular disease.

### 3.3 Methodological quality assessment

**FIGURE 2:**
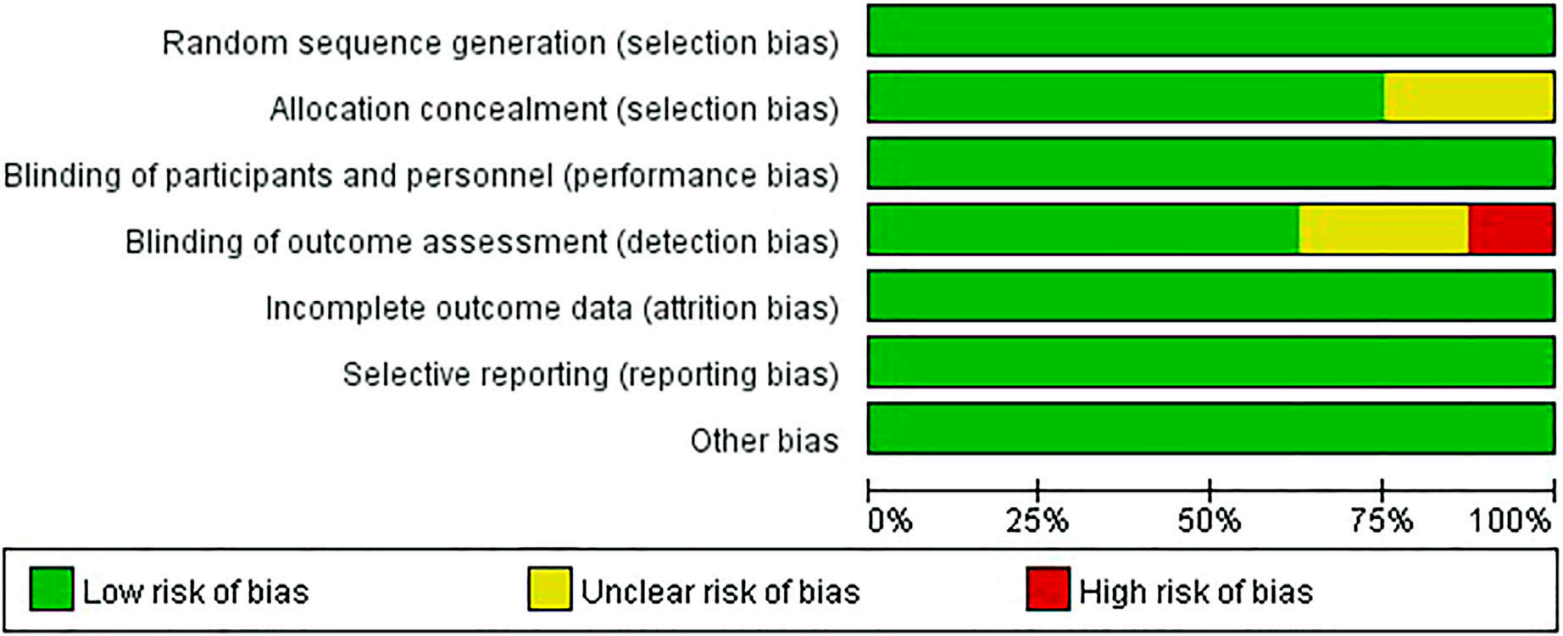
Risk of bias graph.

**FIGURE 3:**
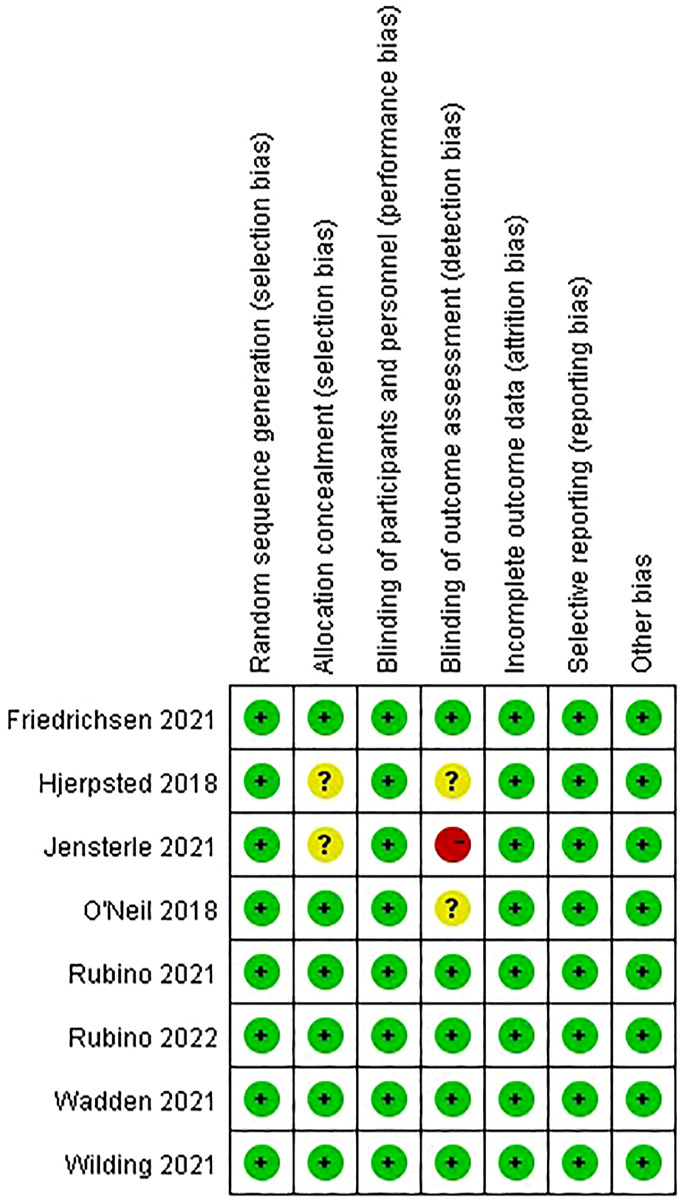
Risk of bias summary.

The risk of bias graph and summary are shown in Figures 2, 3. All studies (Wilding et al., 2021; Wadden et al., 2021; Rubino et al., 2021; Rubino et al., 2022; O'Neil et al., 2018; Friedrichsen et al., 2021; Hjerpsted et al., 2018; Jensterle et al., 2021) were of low risk in terms of random sequence generation, incomplete outcome data, selective reporting, and other bias. One study scored a high risk for blinding of outcome assessment due to the single-blindness in participants (Jensterle et al., 2021), while two studies were double-blind, but the blinding of outcome assessment was unclear (O'Neil et al., 2018; Hjerpsted et al., 2018). In addition, two studies did not mention allocation concealment (Hjerpsted et al., 2018; Jensterle et al., 2021).

### 3.4 Main analyses

#### 3.4.1 Body weight changes

**FIGURE 4:**
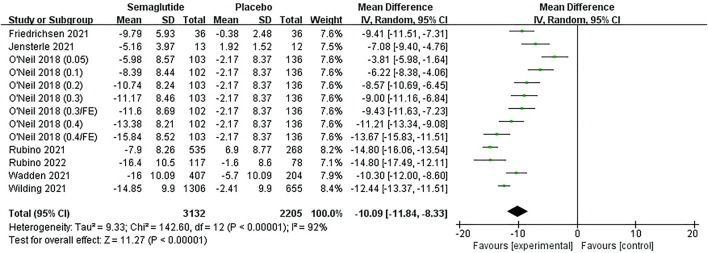
Meta-analysis results of RBW change (%) in included trials.

Seven studies (Wilding et al., 2021; Wadden et al., 2021; Rubino et al., 2021; Rubino et al., 2022; O'Neil et al., 2018; Friedrichsen et al., 2021; Jensterle et al., 2021), including 4,521 individuals, mentioned relative body weight (RBW) and absolute body weight (ABW) changes, five of which were dosed at 2.4 mg/week, one at 1.0 mg/week, and the other by dose escalation. Meta-analysis results revealed the weight loss in semaglutide ranged from 5.16 to 16.40% (5.20–17.36 kg), while the placebo ranged from 0.38 to 5.70% (0.40–6.20 kg) (Figure 4, Supplementary Figure S6, Supplementary Table S3). The heterogeneity test showed statistical heterogeneity among studies (RBW: Ⅰ2 = 92%, *p* ˂ 0.10; ABW: Ⅰ2 = 87%, *p* ˂ 0.10), so random effect models were used for analysis. Combined statistics showed that semaglutide had significantly greater weight loss than placebo, with statistically significant differences (RBW: MD: −10.09%; 95% CI: −11.84 to −8.33; *p* ˂ 0.00001; ABW: MD: −10.54 kg; 95% CI: −12.08 to −9.00; *p* ˂ 0.00001). The funnel plots were also basically symmetric, indicating no publication bias (Supplementary Figures S2A,B).

Subgroup analyses of RBW and ABW changes between semaglutide and placebo based on specific doses were carried out, as shown in Supplementary Tables S4, S5, indicating that the higher the dose, the better the body weight reduction effect. Semaglutide, at any dose, displayed remarkably superiority to the placebo in losing weight.

#### 3.4.2 BMI and WC changes

**FIGURE 5:**
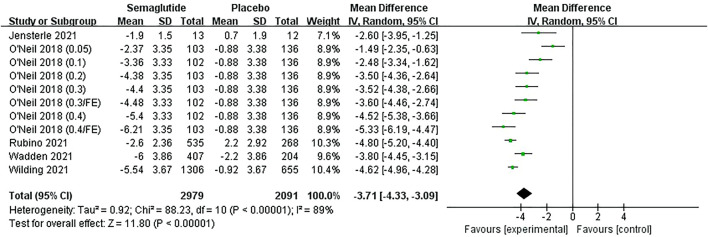
Meta-analysis results of BMI change (kg/m^2^) in included trials.

Five studies (Wilding et al., 2021; Wadden et al., 2021; Rubino et al., 2021; O'Neil et al., 2018; Jensterle et al., 2021), including 4,254 individuals, reported a change in BMI. The meta-analysis results of BMI are shown in Figure 5 and Supplementary Table S3. The heterogeneity test showed statistical heterogeneity among studies (Ⅰ2 = 89%, *p* ˂ 0.10), so a random effect model was adopted for analysis. Combined statistics revealed that semaglutide exhibited a greater reduction in BMI than placebo, with statistically significant differences (MD: −3.71 kg/m^2^; 95% CI: −4.33 to −3.09; *p* ˂ 0.00001). The funnel plot on BMI was also basically symmetric (Supplementary Figure S2C). Subgroup analyses of BMI change revealed that semaglutide 1.0, 2.4, and 2.8 mg led to larger reductions than placebo and more significant with the increase of dose (Supplementary Table S5).

**FIGURE 6:**
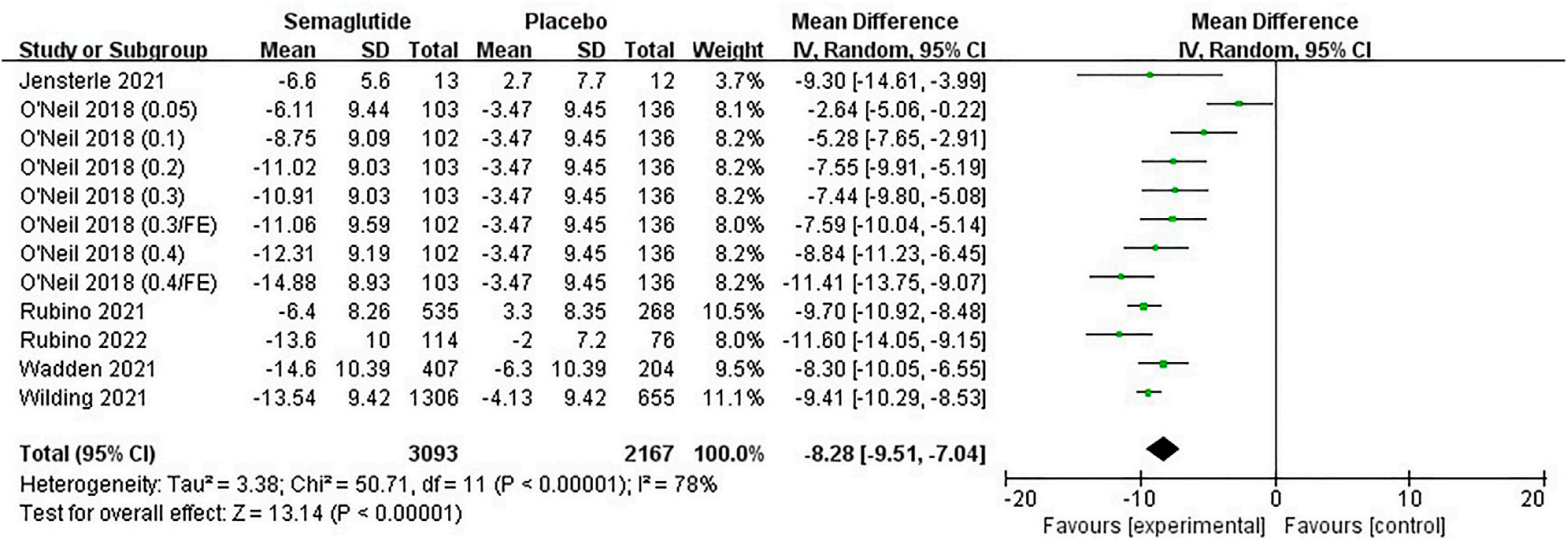
Meta-analysis results of WC change (cm) in included trials.

Six studies (Wilding et al., 2021; Wadden et al., 2021; Rubino et al., 2021; Rubino et al., 2022; O'Neil et al., 2018; Jensterle et al., 2021), including 4,444 individuals, reported a change in WC. The meta-analysis results of WC are shown in Figure 6 and Supplementary Table S3. It was noted that WC reduction in semaglutide ranged from 6.11 to 14.88 cm, while the placebo ranged from 2.00 to 6.30 cm. Statistical heterogeneity existed among studies (Ⅰ^2^ = 78%, *p* ˂ 0.10), and a random effect was applied for analysis. Combined statistics showed that semaglutide tightened WC markedly compared with placebo, with statistically significant differences (MD: −8.28 cm; 95% CI: −9.51 to −7.04; *p* ˂ 0.00001). The funnel plot on WC was also basically symmetric (Supplementary Figure S2D). The subgroup analyses of WC change between semaglutide and placebo were similar to the main meta-analysis results, and the effect was related to the dose too (Supplementary Table S5).

#### 3.4.3 The proportion of participants achieving weight loss of more than 5, 10, 15, and 20%

**FIGURE 7:**
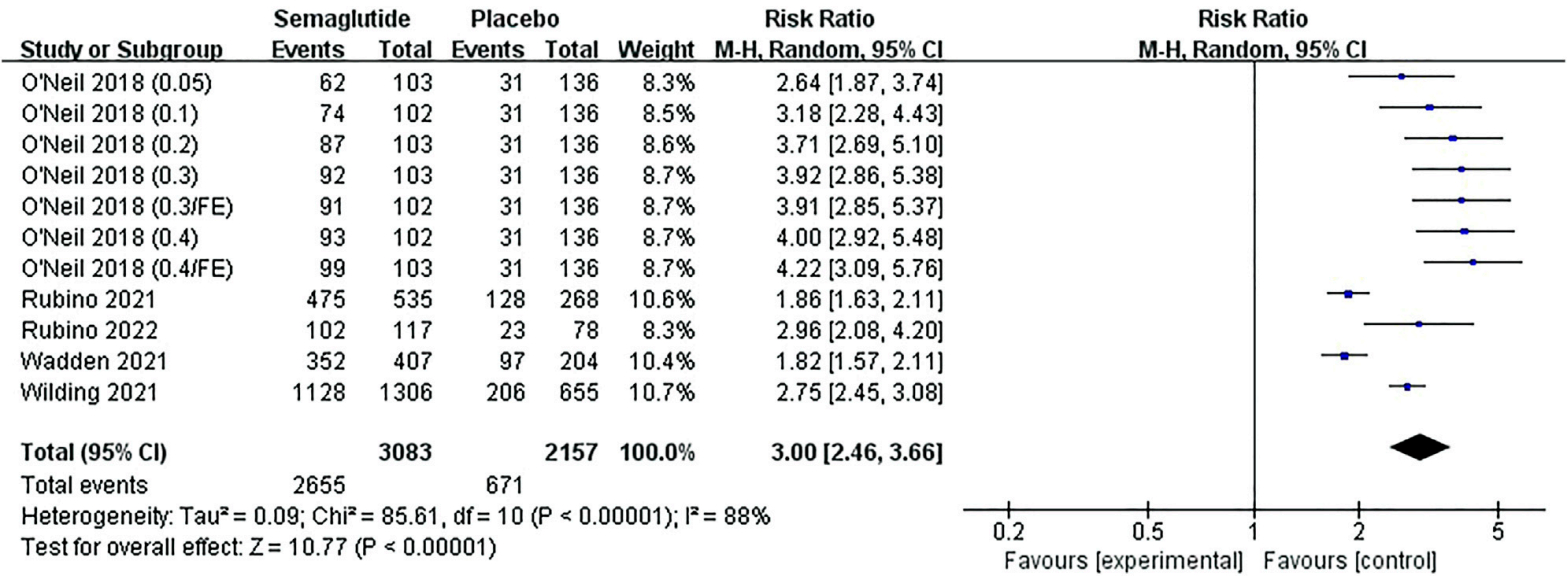
Meta-analysis results of the proportion achieving 5% weight loss in included trials.

Five studies (Wilding et al., 2021; Wadden et al., 2021; Rubino et al., 2021; Rubino et al., 2022; O'Neil et al., 2018), including 4,424 individuals, displayed the proportion of participants who achieved weight loss of more than 5, 10, 15, and 20%. Meta-analysis results can be seen from Figure 7, Supplementary Figures S3–S5 and Supplementary Table S3. There were statistical heterogeneities in the studies of more than 5, 10, and 15% weight loss (5%: Ⅰ^2^ = 88%, *p* ˂ 0.10; 10%: Ⅰ2 = 77%, *p* ˂ 0.10; 15%: Ⅰ2 = 62%, *p* ˂ 0.10). Random effect models were conducted in meta-analyses, which illustrated that the proportion of participants who achieved more than 5, 10, and 15% weight loss were notably higher in semaglutide than that in placebo, and the ratios increased with the proportion of weight loss, with statistically significant differences (5%, RR: 3.00; 95% CI: 2.46 to 3.66; *p* ˂ 0.00001; 10%, RR: 4.85; 95% CI: 3.79 to 6.20; *p* ˂ 0.00001; 15%, RR: 7.99; 95% CI: 5.80 to 11.00; *p* ˂ 0.00001). For the study of more than 20% weight loss, there was no statistical heterogeneity among the studies (Ⅰ2 = 30%, *p* ˃ 0.10); thus, a fixed effect model was selected for analysis, revealing that participants receiving semaglutide were distinctly more likely to achieve more than 20% weight loss from baseline compared with placebo, with statistically significant differences (RR: 11.61; 95% CI: 8.84 to 15.26; *p* ˂ 0.00001).

The corresponding funnel plots were also basically symmetric without publication bias (Supplementary Figure S6). Subgroup analyses referred to weight loss of more than 5, 10, 15, and 20% between semaglutide and placebo based on specific doses were carried out, as shown in Supplementary Tables S4, S5, indicating that the higher the dose (2.8 mg/FE/qw), the greater the proportion achieving correspondent weight loss.

#### 3.4.4 Blood pressure, C-reactive protein, and lipid profiles changes

**FIGURE 8:**
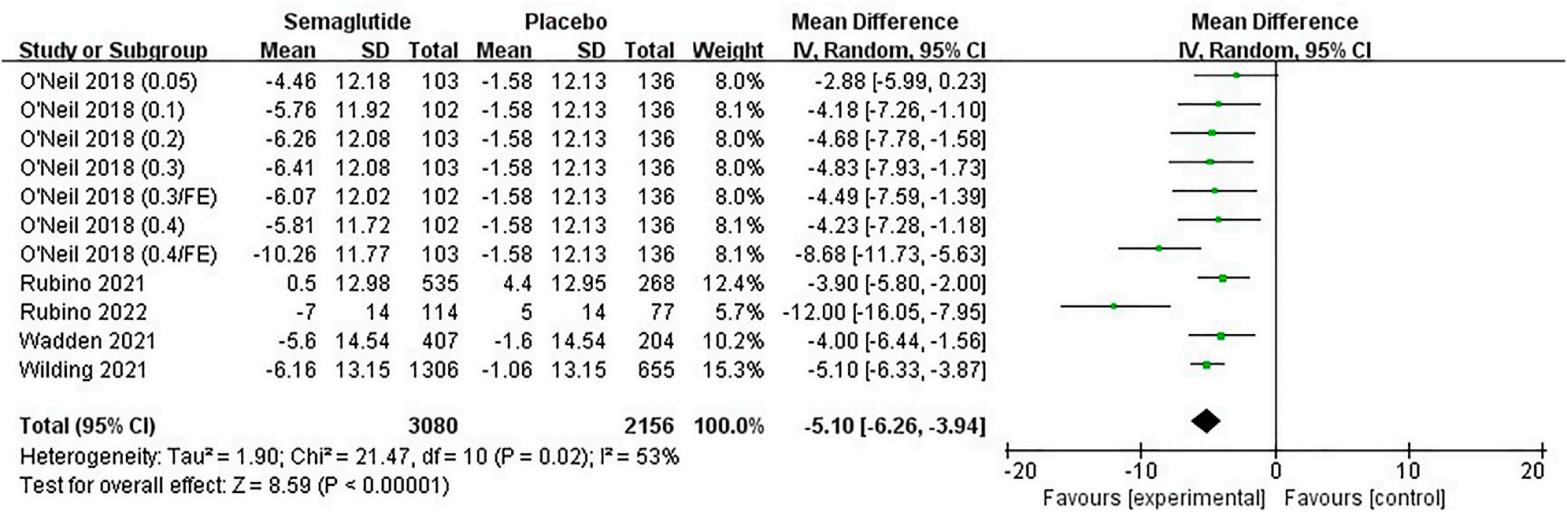
Meta-analysis results of SBP change (mm Hg) in included trials.

Five studies (Wilding et al., 2021; Wadden et al., 2021; Rubino et al., 2021; Rubino et al., 2022; O'Neil et al., 2018), including 4,420 individuals, recorded changes of blood pressure related indicators, SBP and DBP. Meta-analysis results are shown in Figure 8, Supplementary Figure S7, and Supplementary Table S3. Heterogeneity tests of both SBP and DBP showed statistical heterogeneity among studies (SBP:Ⅰ2 = 53%, *p* ˂ 0.10; DBP:Ⅰ2 = 50%, *p* ˂ 0.10); therefore, random effect models were selected. Combined statistical results showed that compared with placebo, semaglutide significantly reduced SBP in obese patients and presented a slight reduction in DBP, with statistically significant differences (SBP, MD: −5.10 mm Hg; 95% CI: −6.26 to −3.94; *p* ˂ 0.00001; DBP, MD: −2.11 mm Hg; 95% CI: −2.89 to −1.32; *p* ˂ 0.00001). Funnel plots were both basically symmetric without publication bias (Supplementary Figures S8A,B).

**FIGURE 9:**
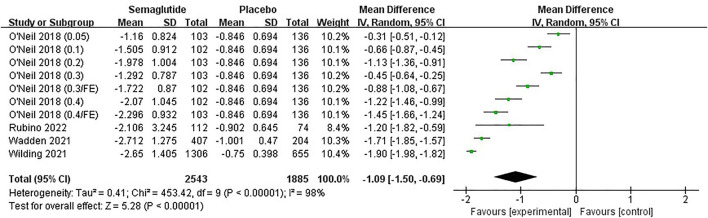
Meta-analysis results of CRP change (mg/L) in included trials.

**FIGURE 10:**
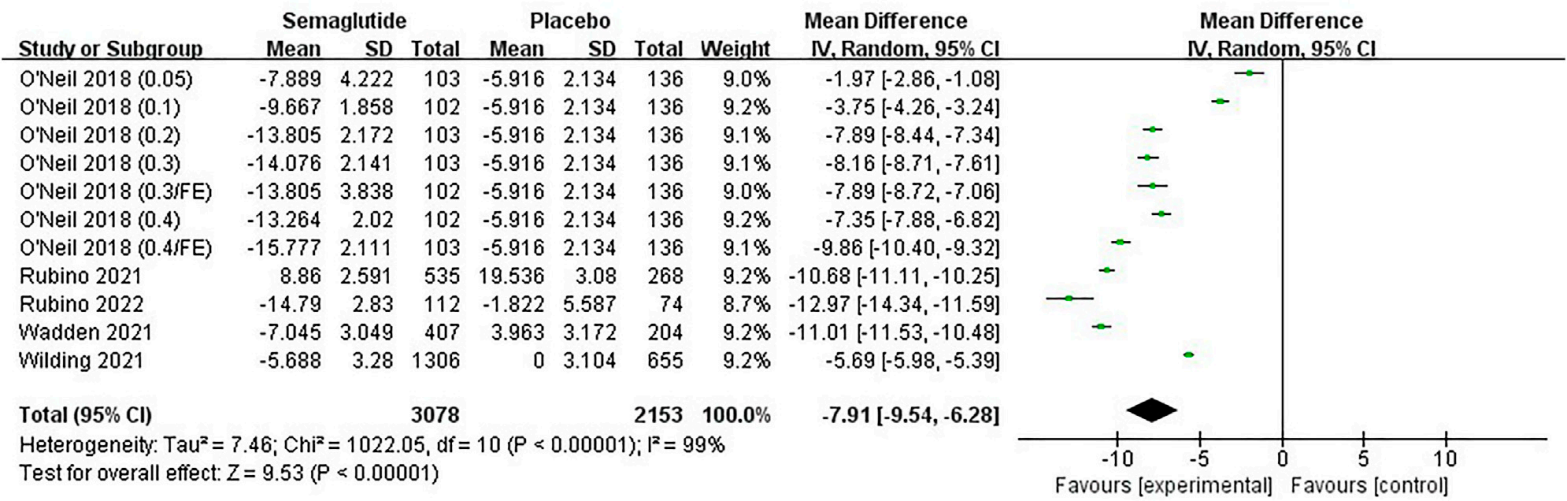
| Meta-analysis results of TC change (mg/dl) in included trials.

Four studies (Wilding et al., 2021; Wadden et al., 2021; Rubino et al., 2022; O'Neil et al., 2018 ), including 3,612 individuals, noted a change in CRP, a marker of inflammation. The analysis result demonstrated that semaglutide achieved a greater decrease from baseline in CRP than placebo, with statistically significant differences (MD: −1.09 mg/L; 95% CI: −1.50 to −0.69; *p* ˂ 0.00001), as shown in Figure 9 and Supplementary Table S3. Five studies (Wilding et al., 2021; Wadden et al., 2021; Rubino et al., 2021; Rubino et al., 2022; O'Neil et al., 2018), including 4,415 individuals, reported changes in TC, HDL, LDL, VLDL, and TG. Four studies (Rubino et al., 2021; Wadden et al., 2021; Wilding et al., 2021; Rubino et al., 2022), including 3,554 individuals, reported a change in FFA. Meta-analyses confirmed that semaglutide was accompanied by numeric improvements in these lipid-related indicators compared with placebo, with the exception of HDL (Figure 10, Supplementary Figures S9–S13, Supplementary Table S3). The corresponding funnel plots were also basically symmetric without publication bias (Supplementary Figure S8C, Supplementary Figure S14).

#### 3.4.5 Adverse events

**FIGURE 11:**
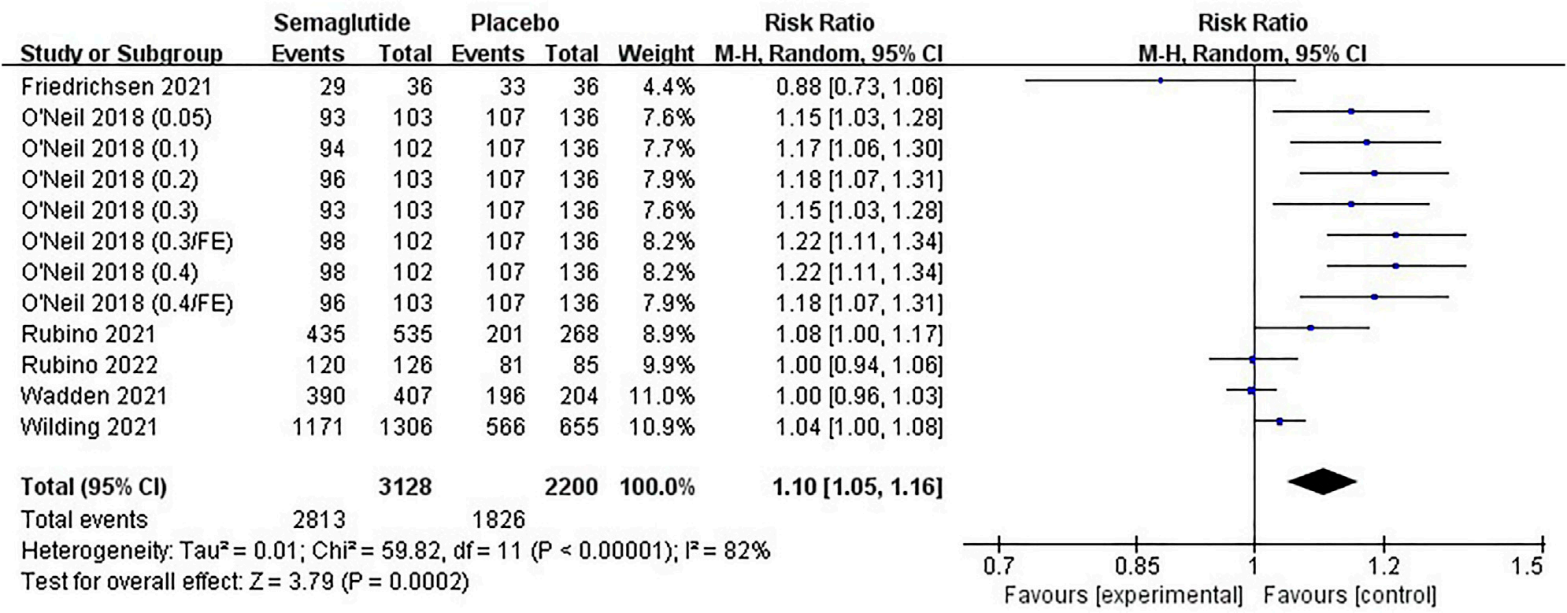
Meta-analysis results of the proportion with AEs in included trials.

Six studies (Wilding et al., 2021; Wadden et al., 2021; Rubino et al., 2021; Rubino et al., 2022; O'Neil et al., 2018; Friedrichsen et al., 2021), including 4,512 individuals, provided the proportion of participants with AEs and SAEs. Eight studies (Wilding et al., 2021; Wadden et al., 2021; Rubino et al., 2021; Rubino et al., 2022; O'Neil et al., 2018; Friedrichsen et al., 2021; Hjerpsted et al., 2018; Jensterle et al., 2021), including 4,567 individuals, reported the proportion of participants with DAEs. Due to heterogeneity (Ⅰ2 = 82%, *p* ˂ 0.10), random effect meta-analysis was conducted for AEs. There was no statistical heterogeneity in the studies of SAEs and DAEs (Ⅰ2 = 8%, *p* ˃ 0.10 and Ⅰ^2^ = 0%, *p* ˃ 0.10); therefore, fixed effect models were selected for meta-analyses. Combined statistics noted that semaglutide increased the incidences of AEs, SAEs, and DAEs compared with placebo, especially DAEs, with statistically significant differences (AEs, RR: 1.10; 95% CI: 1.05 to 1.16; *p* ˂ 0.00001; SAEs, RR: 1.34; 95% CI: 1.10 to 1.65; *p* ˂ 0.00001; DAEs, RR: 2.29; 95% CI: 1.74 to 3.01; *p* ˂ 0.00001), as shown in Figure 11, Supplementary Figures S15, S16 and Supplementary Table S3.

We analyzed several common side effects of semaglutide, such as hypoglycemia and gastrointestinal reactions. Five studies (Wilding et al., 2021; Wadden et al., 2021; Rubino et al., 2021; Rubino et al., 2022; O'Neil et al., 2018), including 4,440 individuals, provided the proportion of participants with hypoglycemia. Seven studies (Wilding et al., 2021; Wadden et al., 2021; Rubino et al., 2021; Rubino et al., 2022; O'Neil et al., 2018; Friedrichsen et al., 2021; Jensterle et al., 2021), including 4,537 individuals, reported the proportion of participants with nausea and diarrhea. As shown in Supplementary Figures S17–S19 and Supplementary Table S3, there were no statistical heterogeneities in the studies, and fixed effect models were selected for analysis, which illustrated that there was no difference in hypoglycemia between semaglutide and placebo (RR: 0.94; 95% CI: 0.66 to 1.34; *p* ˂ 0.00001). Nevertheless, the incidences of nausea and diarrhea were significantly higher in semaglutide than that in placebo, with statistically significant differences (nausea, RR: 2.58; 95% CI: 2.33 to 2.86; *p* ˂ 0.00001; diarrhea, RR: 2.01; 95% CI: 1.79 to 2.27; *p* ˂ 0.00001).

The corresponding funnel plots were basically symmetric (Supplementary Figures S20, S21). Subgroup analyses of semaglutide in different dosages demonstrated that the occurrence of AEs, nausea, and diarrhea were more common in semaglutide 1.0 and 2.8 mg but less in 2.4 mg (Supplementary Table S5).

### 3.5 Sensitivity analysis

The reason for statistical heterogeneity might be inconsistent dose and duration of administration between studies. Sensitivity analyses of two main outcomes, namely, RBW change and the proportion achieving 5% weight loss, were carried out through Stata 14, as shown in Supplementary Figures S2, S23. Slight heterogeneities in RBW change and 5% weight loss were yielded after deleting any of the studies. Hence, meta-regression analyses were presented to explore the heterogeneities in different baseline variables, including dose, female ratio, duration, race, and comorbidities (Supplementary Table S6). Except the dose in 5% weight loss, other variables did not exhibit correlation with the RBW change and 5% weight loss, suggesting stable and reliable results with dose-dependence in this research.

## 4 Discussion

Our systematic review of eight studies indicated that semaglutide showed an attractive weight loss effect in obese or overweight patients without diabetes. Compared with placebo, semaglutide administered subcutaneously reduced body weight by 10.09% (10.54 kg), BMI by 3.71 kg/m^2^, and WC by 8.28 cm, achieved more than 5, 10, 15, and 20% weight loss with a higher proportion of participants, and exhibited certainly positive effects on blood pressure, CRP, and lipid profiles. Additionally, semaglutide had more adverse effects than placebo, especially nausea and diarrhea, but they were transient and mild-to-moderate. Subgroup analysis revealed semaglutide 1.0, 2.4, and 2.8 mg led to distinct reduction in body weight, BMI, and WC, but exhibited more events in semaglutide 2.8 mg, whereas less in 2.4 mg. All results were stable and reliable.

A series of reviews elaborated on the efficacy and safety of semaglutide in obese patients with or without diabetes, generally containing weight-related changes (Tan et al., 2017; Li et al., 2018; Davies et al., 2021; Li et al., 2021; He et al., 2022). The previous studies were almost referred to patients with or without diabetes and the comparison between 2.4 mg-dosed semaglutide and placebo. In order to facilitate clinical application, semaglutide should be investigated as a conventional weight-lowering drug for normal obese patients without diabetes, and the weight loss effect should be evaluated by different doses; furthermore, the potentially beneficial effects could be sought. By and large, our study was considered to be the latest and most comprehensive systematic review of randomized controlled trials, which fully examined the weight loss effects of different dosages of semaglutide compared with placebo in obese or overweight patients without diabetes. The novel and high-quality clinical trial data published in top authoritative journals provided powerful evidence for our meta-analysis (Wilding et al., 2021; Wadden et al., 2021; Rubino et al., 2021; Rubino et al., 2022; O'Neil et al., 2018). The other three studies did not directly explore the weight loss effect in obese or overweight patients without diabetes, but demonstrated it in other ways, such as reducing appetite and energy intake, delaying gastric emptying, and decreasing local fat accumulation, respectively (Hjerpsted et al., 2018; Friedrichsen et al., 2021; Jensterle et al., 2021).

GLP-1RA was initially approved for the treatment of type 2 diabetes, surprisingly exhibiting a distinct weight loss effect while controlling blood glucose (Monami et al., 2012; Xu et al., 2020). GLP-1RA has been reported to reduce body weight through a variety of pathways, including inhibiting gastrointestinal peristalsis and gastric secretion, prolonging gastric emptying, lessening energy intake, as well as generating satiety and suppressing appetite via the central nervous system, especially the hypothalamus (Zander et al., 2002; Drucker 2018). Compared to the approved liraglutide, once-weekly semaglutide elicited greater weight loss according to the studies ( Rubino et al., 2022; O'Neil et al., 2018), which may be possibly due to the difference in energy intake regulation; that is, semaglutide may be associated with reduced food craving, while liraglutide was less obvious (Gabery et al., 2020). Currently, the marketed GLP-1RA, including once-daily liraglutide (O'Neil et al., 2018; Nauck et al., 2016; Rubino et al., 2022), approved for non-diabetic obesity, once-weekly exenatide (Su et al., 2016; Ahmann et al., 2018), and dulaglutide (Pratley et al., 2018; Bonora et al., 2021) which have not been approved yet, cannot achieve the same effect as semaglutide in weight loss (O'Neil et al., 2018; Nauck et al., 2016; Rubino et al., 2022; Ahmann et al., 2018; Su et al., 2016; Pratley et al., 2018; Bonora et al., 2021).

Semaglutide, a new once-weekly GLP-1RA, has performed a significant weight loss effect in obese or overweight patients with or without diabetes (Blundell et al., 2017; Ahrén et al., 2018; Kushner et al., 2020; Davies et al., 2021; Kadowaki et al., 2022), and exhibited favorable advantages in reducing obesity complications (Ryan et al., 2020; Kushner et al., 2021). Iacobellis and Villasnte Fricke 2020 reported that semaglutide decreased epicardial adipose tissue thickness by almost 20% after 12 weeks in subjects with type 2 diabetes and obesity, suggesting a reduction in cardiometabolic risk. Ji et al. (2021) revealed that semaglutide was related to a significant 26% reduction in the risk of major adverse cardiovascular events compared with placebo, with a 39% reduction in stroke. Ryan et al. (2020) evaluated the superiority of semaglutide on cardiovascular event reduction in people with overweight or obesity, which was implicated as an anti-inflammatory mechanism. It is well known that CRP is not only a non-specific inflammatory marker, but also directly participates in cardiovascular diseases such as inflammation and atherosclerosis, and is the most powerful predictor and risk factor for cardiovascular diseases. In our meta-analysis, semaglutide showed a significant reduction in SBP and CRP and lowered the levels of lipid-related indicators in obese patients, which exhibited certainly positive effects on metabolic syndromes such as dyslipidemia, hypertension, obstructive sleep apnea, and cardiovascular disease. In this regard, it is successfully recognized that semaglutide may be particularly suitable for obese patients with cardiovascular disease.

Compared with placebo, semaglutide increased the incidences of AEs, SAEs, and DAEs, especially DAEs, and gastrointestinal side effects accounted for the majority, particularly nausea and diarrhea, which is probably owing to the long-term gastric emptying (Aroda et al., 2019; Wharton et al., 2022). Since DAEs occurred more frequently in semaglutide, it may be due to its longer half-life (165 h), potentially inducing a more abrupt return of hunger when pausing the treatment and leading to permanent discontinuations (Lau et al., 2015; Rubino et al., 2022). Intriguingly, there was no difference in hypoglycemia between semaglutide and placebo. It is worth noting that weight loss observed with semaglutide was mainly mediated by therapeutic effect, rather than the occurrence of those adverse events (Lingvay et al., 2020), indicating that semaglutide can be regarded as a weight management agent with generally acceptable safety.

This study has several important strengths. First, the latest and most comprehensive systematic review examining the weight loss effects of semaglutide was totally conducted in obese or overweight patients without diabetes, which is expected to provide favorable strategies for normal obese patients as a conventional weight-lowering drug. Second, we outlined the correlation between baseline variables and main effects, proposing the influence of dose on weight loss effects and adverse events, further providing direction for clinical individualized medication. Third, semaglutide elaborated potentially beneficial effects on cardiovascular disease by controlling blood pressure, inflammation, and lipids, indicating the additional cardioprotective effects beyond weight reduction in obese people without diabetes.

Limitations also existed in our study. First, because of the relatively limited number of studies about semaglutide treating obesity without diabetes, one of our studies was divided into seven separate trials for analyses, which might affect the homogeneity and publication bias. Second, previous studies based on semaglutide in obese patients with or without diabetes had been published, potentially affecting the innovation of our research; thus, some other aspects were discussed, such as the narrowing of patients, the specific dose of semaglutide, and the potentially beneficial effects on cardiovascular disease. Third, despite subgroup and meta-regression analyses being conducted to explore the heterogeneities in different baseline variables (Supplementary Tables S4–S6), high heterogeneity remained in those main outcomes. However, taking weight loss effect and safety into full consideration, the recommended dosage of semaglutide of 2.4 mg once weekly was suggested in our study according to the drug instruction (Shi et al., 2022), which exhibited higher efficacy outcomes and offered an effective dosing strategy for obese patients.

At present, semaglutide is mainly available in two formulations, once-weekly subcutaneous semaglutide (Ozempic^®^, 0.5 mg or 1.0 mg; Wegovy^®^, 2.4 mg) and once-daily oral semaglutide (Rybelsus^®^, 7 mg or 14 mg) (Meier 2021; Zhong et al., 2021), the first oral GLP-1RA in the world, which provides effective glycemic control in type 2 diabetes when combined with diet and exercise intervention, bringing great convenience to patients (Davies et al., 2017; Gibbons et al., 2021). We hope that more clinical trials will be conducted in the future to evaluate the weight loss effects of oral semaglutide so as to provide a more convenient, effective, and safe treatment for the majority of obese patients.

## 5 Conclusion

In conclusion, semaglutide can significantly reduce weight and BMI for obese or overweight patients without diabetes and has a positive effect on heart-related risk factors such as waist circumference, blood pressure, CRP, and lipid profiles with acceptable safety and high compliance, which can effectively improve obese patients’ health and quality of life.

## Data Availability

The original contributions presented in the study are included in the article/Supplementary Material; further inquiries can be directed to the corresponding authors.
