# Supplementary material for: Efficacy and safety of semaglutide on weight loss in obese or overweight patients without diabetes: A systematic review and meta-analysis of randomized controlled trials
Source: Front Pharmacol. 2022 Sep 14;13:935823. doi: 10.3389/fphar.2022.935823 (PMC9515581; doi:10.3389/fphar.2022.935823)
Supplement: Supplementary file 1 [file DataSheet1.DOCX]

Supplementary Material

# Supplementary Tables

Table S1. Search strategies

| Databases | Search strategies |
| --- | --- |
| PubMed | ((("Obesity"[Mesh]) OR ((((((obese[Title/Abstract]) OR (fat[Title/Abstract])) OR (obe*[Title/Abstract])) OR (adiposity[Title/Abstract])) OR (overweight[Title/Abstract])) OR (over weight[Title/Abstract]))) AND (("semaglutide" [Supplementary Concept]) OR ((rybelsus[Title/Abstract]) OR (Ozempic[Title/Abstract])))) AND ((randomized controlled trial[Publication Type] OR randomized[Title/Abstract] OR placebo[Title/Abstract])) |
| Cochrane library | #1 MeSH descriptor: [Obesity] explode all trees  #2 (obese):ti,ab,kw OR (fat):ti,ab,kw OR (obe*):ti,ab,kw OR (adiposity):ti,ab,kw OR (overweight):ti,ab,kw OR (over weight):ti,ab,kw (Word variations have been searched)  #3 #1 OR #2  #4 MeSH descriptor: [] explode all trees  #5 (semaglutide):ti,ab,kw OR (rybelsus):ti,ab,kw OR (Ozempic):ti,ab,kw (Word variations have been searched)  #6 #4 OR #5  #7 #3 AND #6 |
| EMBASE | #8. #3 AND #6 AND #7  #7. 'randomized controlled trial':ab,ti OR randomized:ab,ti OR placebo:ab,ti  #6. #4 OR #5  #5. rybelsus:ab,ti OR ozempic:ab,ti  #4. 'semaglutide'/exp OR 'semaglutide'  #3. #1 OR #2  #2. obese:ab,ti OR fat:ab,ti OR obe*:ab,ti OR adiposity:ab,ti OR overweight:ab,ti OR 'over weight':ab,ti  #1. 'obesity'/exp |
| ClinicalTrials.gov | 55 Studies found for: Interventional Studies \| Obesity \| Semaglutide |

Table S2. Different proportions of individuals with or without comorbidities in included studies.

| Study ID | Intervention and comparison | BMI (kg/m^2^) | Types of comorbidities (%) | | | | Without comorbidities (%) |
| --- | --- | --- | --- | --- | --- | --- | --- |
|  |  |  | Dyslipidemia | Hypertension | Obstructive sleep apnea | Coronary artery disease |  |
| Wilding 2021 | Semaglutide | ≥ 30 or ≥ 27 with 1 or more comorbidities | 38.2 | 36.1 | 12.2 | 2.5 | 25.1 |
|  | Placebo |  | 34.5 | 35.7 | 10.8 | 2.6 | 24.9 |
| Wadden 2021 | Semaglutide | ≥ 30 or ≥ 27 with 1 or more comorbidities | 35.6 | 35.6 | 14.3 | 1.5 | 24.3 |
|  | Placebo |  | 32.8 | 32.8 | 9.3 | 2.0 | 24.0 |
| Rubino 2021 | Semaglutide | ≥ 30 or ≥ 27 with 1 or more comorbidities | 35.3 | 37.2 | 11.4 | 0.7 | 26.9 |
|  | Placebo |  | 36.9 | 36.9 | 12.3 | 1.1 | 26.1 |
| Rubino 2022 | Semaglutide | ≥ 30 or ≥ 27 with 1 or more comorbidities | 47.6 | 38.1 | 19.0 | 3.2 | 25.4 |
|  | Placebo |  | 42.4 | 45.9 | 22.4 | 4.7 | 18.8 |
| O'Neil 2018 | Semaglutide | ≥ 30 | NA | NA | NA | NA | 100 |
|  | Placebo |  | NA | NA | NA | NA | 100 |
| Friedrichsen 2021 | Semaglutide | 30~45 | NA | NA | NA | NA | 100 |
|  | Placebo |  | NA | NA | NA | NA | 100 |
| Hjerpsted 2018 | Semaglutide | 30~45 | NA | NA | NA | NA | 100 |
|  | Placebo |  | NA | NA | NA | NA | 100 |
| Jensterle 2021 | Semaglutide | ＞ 30 | NA | NA | NA | NA | 100 |
|  | Placebo |  | NA | NA | NA | NA | 100 |

Table S3. Comparison of outcome endpoints between semaglutide and placebo.

| **Outcome** | **Studies** | **Participants** | **Statistical Method** | **Effect Estimate** |
| --- | --- | --- | --- | --- |
| **Continuous data** |  |  |  |  |
| 1.1 RBW | 7 | 4521 | Mean Difference (IV, Random, 95% CI) | -10.09 [-11.84, -8.33] |
| 1.2 ABW | 7 | 4521 | Mean Difference (IV, Random, 95% CI) | -10.54 [-12.08, -9.00] |
| 1.3 BMI | 5 | 4254 | Mean Difference (IV, Random, 95% CI) | -3.71 [-4.33, -3.09] |
| 1.4 WC | 6 | 4444 | Mean Difference (IV, Random, 95% CI) | -8.28 [-9.51, -7.04] |
| 1.5 SBP | 5 | 4420 | Mean Difference (IV, Random, 95% CI) | -5.10 [-6.26, -3.94] |
| 1.6 DBP | 5 | 4420 | Mean Difference (IV, Random, 95% CI) | -2.11 [-2.89, -1.32] |
| 1.7 CRP | 4 | 3612 | Mean Difference (IV, Random, 95% CI) | -1.09 [-1.50, -0.69] |
| 1.8 Total Cholesterol | 5 | 4415 | Mean Difference (IV, Random, 95% CI) | -7.91 [-9.54, -6.28] |
| 1.9 HDL cholesterol | 5 | 4413 | Mean Difference (IV, Random, 95% CI) | 0.54 [0.04, 1.05] |
| 1.10 LDL cholesterol | 5 | 4413 | Mean Difference (IV, Random, 95% CI) | -5.39 [-6.69, -4.10] |
| 1.11 VLDL cholesterol | 5 | 4413 | Mean Difference (IV, Fixed, 95% CI) | -3.67 [-4.16, -3.18] |
| 1.12 Triglycerides | 5 | 4413 | Mean Difference (IV, Random, 95% CI) | -15.79 [-18.29, -13.29] |
| 1.13 Free fatty acids | 4 | 3554 | Mean Difference (IV, Fixed, 95% CI) | -1.12 [-2.22, -0.02] |
| **Dichotomous data** |  |  |  |  |
| 2.1 Weight loss 5% | 5 | 4424 | Risk Ratio (M-H, Random, 95% CI) | 3.00 [2.46, 3.66] |
| 2.2 Weight loss 10% | 5 | 4424 | Risk Ratio (M-H, Random, 95% CI) | 4.85 [3.79, 6.20] |
| 2.3 Weight loss 15% | 5 | 4424 | Risk Ratio (M-H, Random, 95% CI) | 7.99 [5.80, 11.00] |
| 2.4 Weight loss 20% | 5 | 4424 | Risk Ratio (M-H, Fixed, 95% CI) | 11.61 [8.84, 15.26] |
| 2.5 AEs | 6 | 4512 | Risk Ratio (M-H, Random, 95% CI) | 1.10 [1.05, 1.16] |
| 2.6 SAEs | 6 | 4512 | Risk Ratio (M-H, Fixed, 95% CI) | 1.34 [1.10, 1.65] |
| 2.7 DAEs | 8 | 4567 | Risk Ratio (M-H, Fixed, 95% CI) | 2.29 [1.74, 3.01] |
| 2.8 Hypoglycemia | 5 | 4440 | Risk Ratio (M-H, Fixed, 95% CI) | 0.94 [0.66, 1.34] |
| 2.9 Nausea | 7 | 4537 | Risk Ratio (M-H, Fixed, 95% CI) | 2.58 [2.33, 2.86] |
| 2.10 Diarrhea | 7 | 4537 | Risk Ratio (M-H, Fixed, 95% CI) | 2.01 [1.79, 2.27] |

Table S4. Subgroup analysis of RBW and weight loss 5% between semaglutide and placebo based on specific doses.

| **Outcome** | **Subgroup** | **Studies** | **Participants** | **Statistical Method** | **Effect Estimate** |
| --- | --- | --- | --- | --- | --- |
| RBW | Semaglutide 0.35 mg/qw vs placebo | 1 | 239 | Mean Difference (IV, Random, 95% CI) | -3.81 [-5.98, -1.64] |
|  | Semaglutide 0.7 mg/qw vs placebo | 1 | 238 | Mean Difference (IV, Random, 95% CI) | -6.22 [-8.38, -4.06] |
|  | Semaglutide 1.0 mg/qw vs placebo | 1 | 25 | Mean Difference (IV, Random, 95% CI) | -7.08 [-9.40, -4.76] |
|  | Semaglutide 1.4 mg/qw vs placebo | 1 | 239 | Mean Difference (IV, Random, 95% CI) | -8.57 [-10.69, -6.45] |
|  | Semaglutide 2.1 mg/qw vs placebo | 1 | 239 | Mean Difference (IV, Random, 95% CI) | -9.00 [-11.16, -6.84] |
|  | Semaglutide 2.1 mg/FE/qw vs placebo | 1 | 238 | Mean Difference (IV, Random, 95% CI) | -9.43 [-11.63, -7.23] |
|  | Semaglutide 2.4 mg/qw vs placebo | 5 | 3642 | Mean Difference (IV, Random, 95% CI) | -12.34 [-14.26, -10.42] |
|  | Semaglutide 2.8 mg/qw vs placebo | 1 | 238 | Mean Difference (IV, Random, 95% CI) | -11.21 [-13.34, -9.08] |
|  | Semaglutide 2.8 mg/FE/qw vs placebo | 1 | 239 | Mean Difference (IV, Random, 95% CI) | -13.67 [-15.83, -11.51] |
| Weight loss 5% | Semaglutide 0.35 mg/qw vs placebo | 1 | 239 | Risk Ratio (M-H, Random, 95% CI) | 2.64 [1.87, 3.74] |
|  | Semaglutide 0.7 mg/qw vs placebo | 1 | 238 | Risk Ratio (M-H, Random, 95% CI) | 3.18 [2.28, 4.43] |
|  | Semaglutide 1.4 mg/qw vs placebo | 1 | 239 | Risk Ratio (M-H, Random, 95% CI) | 3.71 [2.69, 5.10] |
|  | Semaglutide 2.1 mg/qw vs placebo | 1 | 239 | Risk Ratio (M-H, Random, 95% CI) | 3.92 [2.86, 5.38] |
|  | Semaglutide 2.1 mg/FE/qw vs placebo | 1 | 238 | Risk Ratio (M-H, Random, 95% CI) | 3.91 [2.85, 5.37] |
|  | Semaglutide 2.4 mg/qw vs placebo | 4 | 3570 | Risk Ratio (M-H, Random, 95% CI) | 2.25 [1.74, 2.89] |
|  | Semaglutide 2.8 mg/qw vs placebo | 1 | 238 | Risk Ratio (M-H, Random, 95% CI) | 4.00 [2.92, 5.48] |
|  | Semaglutide 2.8 mg/FE/qw vs placebo | 1 | 239 | Risk Ratio (M-H, Random, 95% CI) | 4.22 [3.09, 5.76] |

Table S5. Subgroup analysis of some main results between semaglutide and placebo based on three doses.

| **Outcome** | **Subgroup** | **Studies** | **Participants** | **Statistical Method** | **Effect Estimate** |
| --- | --- | --- | --- | --- | --- |
| RBW | Semaglutide 1.0 mg/qw vs placebo | 1 | 239 | Mean Difference (IV, Random, 95% CI) | -7.08 [-9.40, -4.76] |
|  | Semaglutide 2.4 mg/qw vs placebo | 5 | 3642 | Mean Difference (IV, Random, 95% CI) | -12.34 [-14.26, -10.42] |
|  | Semaglutide 2.8 mg/FE/qw vs placebo | 1 | 239 | Mean Difference (IV, Random, 95% CI) | -13.67 [-15.83, -11.51] |
| ABW | Semaglutide 1.0 mg/qw vs placebo | 1 | 25 | Mean Difference (IV, Random, 95% CI) | -7.10 [-9.43, -4.77] |
|  | Semaglutide 2.4 mg/qw vs placebo | 5 | 3642 | Mean Difference (IV, Random, 95% CI) | -12.20 [-13.47, -10.94] |
|  | Semaglutide 2.8 mg/FE/qw vs placebo | 1 | 239 | Mean Difference (IV, Random, 95% CI) | -14.88 [-17.30, -12.46] |
| BMI | Semaglutide 1.0 mg/qw vs placebo | 1 | 25 | Mean Difference (IV, Random, 95% CI) | -2.60 [-3.95, -1.25] |
|  | Semaglutide 2.4 mg/qw vs placebo | 3 | 3375 | Mean Difference (IV, Random, 95% CI) | -4.48 [-4.95, -4.00] |
|  | Semaglutide 2.8 mg/FE/qw vs placebo | 1 | 239 | Mean Difference (IV, Random, 95% CI) | -5.33 [-6.19, -4.47] |
| WC | Semaglutide 1.0 mg/qw vs placebo | 1 | 25 | Mean Difference (IV, Fixed, 95% CI) | -9.30 [-14.61, -3.99] |
|  | Semaglutide 2.4 mg/qw vs placebo | 4 | 3565 | Mean Difference (IV, Fixed, 95% CI) | -9.49 [-10.13, -8.85] |
|  | Semaglutide 2.8 mg/FE/qw vs placebo | 1 | 239 | Mean Difference (IV, Fixed, 95% CI) | -11.41 [-13.75, -9.07] |
| Weight loss 5% | Semaglutide 2.4 mg/qw vs placebo | 4 | 3570 | Risk Ratio (M-H, Random, 95% CI) | 2.25 [1.74, 2.89] |
|  | Semaglutide 2.8 mg/FE/qw vs placebo | 1 | 239 | Risk Ratio (M-H, Random, 95% CI) | 4.22 [3.09, 5.76] |
| Weight loss 10% | Semaglutide 2.4 mg/qw vs placebo | 4 | 3570 | Risk Ratio (M-H, Random, 95% CI) | 4.07 [2.83, 5.85] |
|  | Semaglutide 2.8 mg/FE/qw vs placebo | 1 | 239 | Risk Ratio (M-H, Random, 95% CI) | 7.55 [4.55, 12.52] |
| Weight loss 15% | Semaglutide 2.4 mg/qw vs placebo | 4 | 3570 | Risk Ratio (M-H, Random, 95% CI) | 6.97 [4.42, 10.98] |
|  | Semaglutide 2.8 mg/FE/qw vs placebo | 1 | 239 | Risk Ratio (M-H, Random, 95% CI) | 17.17 [7.17, 41.09] |
| Weight loss 20% | Semaglutide 2.4 mg/qw vs placebo | 4 | 3570 | Risk Ratio (M-H, Random, 95% CI) | 11.55 [7.27, 18.36] |
|  | Semaglutide 2.8 mg/FE/qw vs placebo | 1 | 239 | Risk Ratio (M-H, Random, 95% CI) | 9.24 [2.83, 30.15] |
| AEs | Semaglutide 2.4 mg/qw vs placebo | 5 | 3658 | Risk Ratio (M-H, Fixed, 95% CI) | 1.03 [1.01, 1.06] |
|  | Semaglutide 2.8 mg/FE/qw vs placebo | 1 | 239 | Risk Ratio (M-H, Fixed, 95% CI) | 1.18 [1.14, 1.23] |
| Nausea | Semaglutide 1.0 mg/qw vs placebo | 1 | 25 | Risk Ratio (M-H, Fixed, 95% CI) | 8.31 [1.23, 56.17] |
|  | Semaglutide 2.4 mg/qw vs placebo | 5 | 3658 | Risk Ratio (M-H, Fixed, 95% CI) | 2.61 [2.28, 2.99] |
|  | Semaglutide 2.8 mg/FE/qw vs placebo | 1 | 239 | Risk Ratio (M-H, Fixed, 95% CI) | 2.75 [1.82, 4.16] |
| Diarrhea | Semaglutide 1.0 mg/qw vs placebo | 1 | 25 | Risk Ratio (M-H, Fixed, 95% CI) | 8.31 [1.23, 56.17] |
|  | Semaglutide 2.4 mg/qw vs placebo | 5 | 3658 | Risk Ratio (M-H, Fixed, 95% CI) | 1.85 [1.60, 2.13] |
|  | Semaglutide 2.8 mg/FE/qw vs placebo | 1 | 239 | Risk Ratio (M-H, Fixed, 95% CI) | 2.31 [1.32, 4.04] |

Table S6. Meta-regression analysis of some main results between semaglutide and placebo.

| **Outcome** | **Independent variable** | **Coefficient** | **Standard error** | **t** | **P-value** | **95% CI** | |
| --- | --- | --- | --- | --- | --- | --- | --- |
| RBW | dose | 0.6916235 | 0.0866630 | -2.94 | 0.013 | 0.5249222 | 0.9112648 |
|  | female ratio | 0.5107916 | 0.1385003 | -2.48 | 0.031 | 0.2812287 | 0.9277433 |
|  | duration | 0.6568068 | 0.0914169 | -3.02 | 0.012 | 0.4834985 | 0.8922368 |
|  | race | 0.7549919 | 0.0769940 | -2.76 | 0.019 | 0.6032012 | 0.9449795 |
|  | comorbidities | 0.8421627 | 0.2393242 | -0.60 | 0.558 | 0.4505646 | 1.5741100 |
| Weight loss 5% | dose | 0.9860868 | 0.1834584 | -0.08 | 0.942 | 0.6473435 | 1.5020880 |
|  | duration | 0.4727868 | 0.0755416 | -4.69 | 0.001 | 0.3293754 | 0.6786400 |
|  | race | 0.6526488 | 0.1050870 | -2.65 | 0.026 | 0.4534092 | 0.9394392 |
|  | comorbidities | 0.4727868 | 0.0755416 | -4.69 | 0.001 | 0.3293754 | 0.6786400 |

# Supplementary Figures


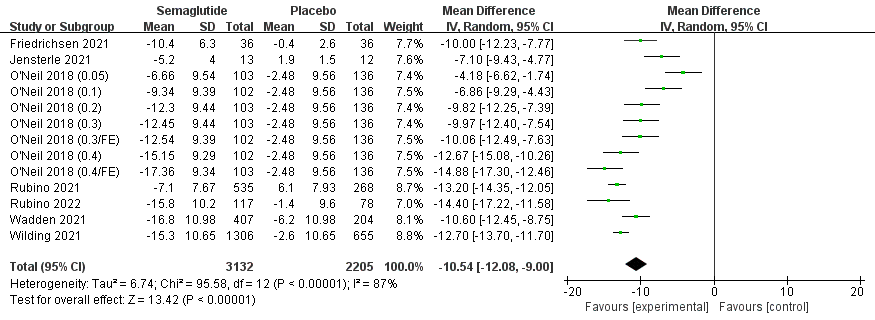


Figure S1. Meta-analysis results of ABW change (kg) in included trials.


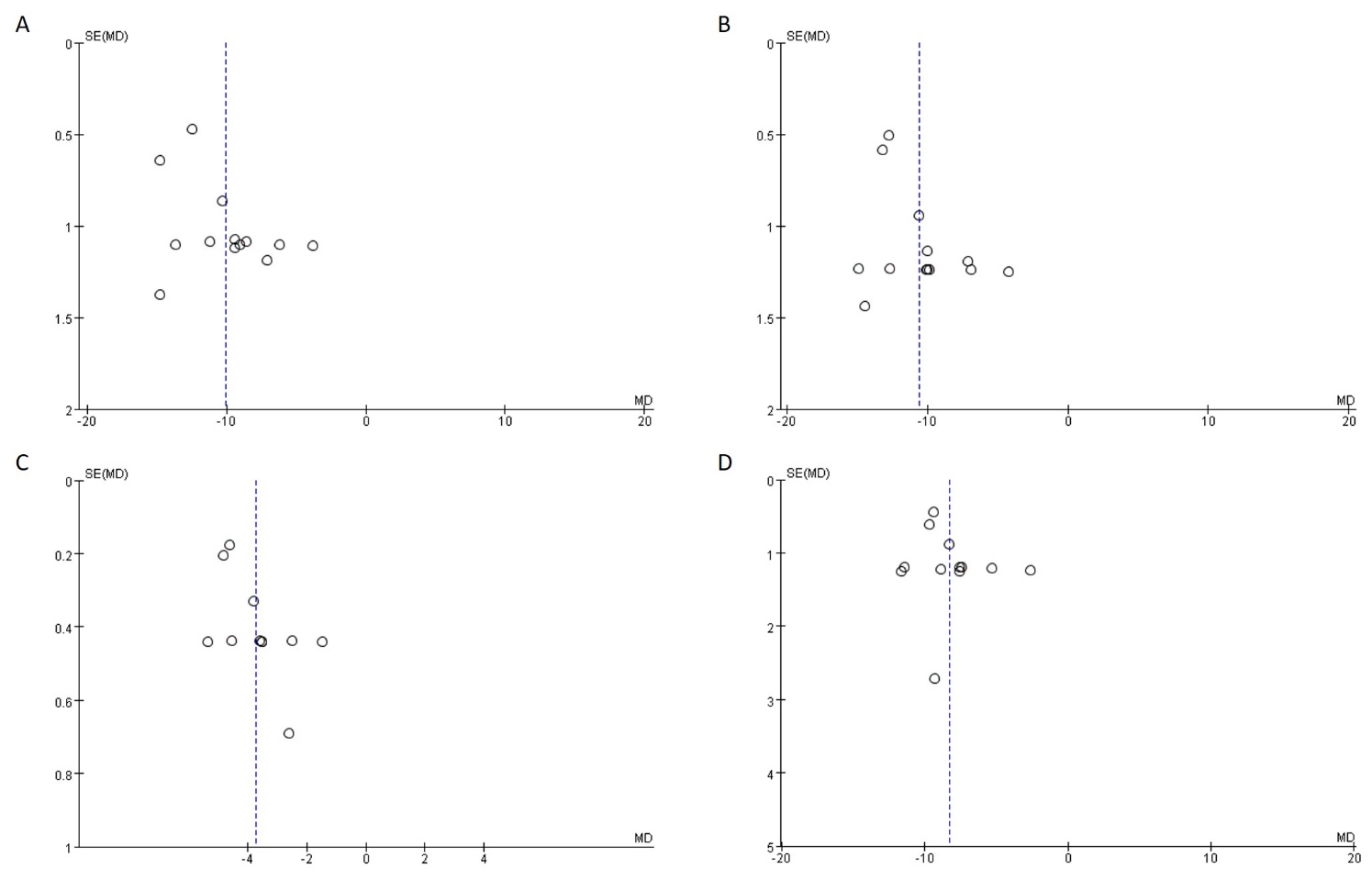


Figure S2. Funnel plots of RBW change (%) (A), ABW change (kg) (B), BMI change (kg/m^2^) (C) and WC change (cm) (D) weight loss in included trials.


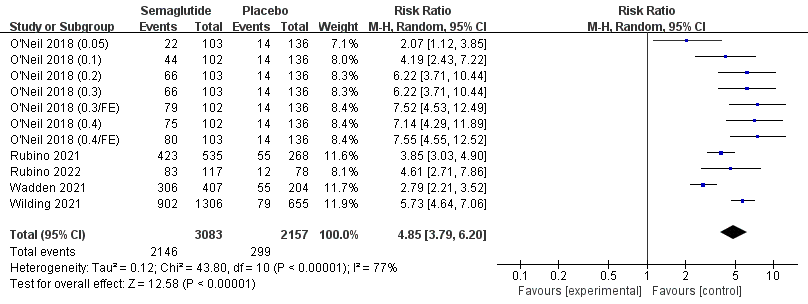


Figure S3. Meta-analysis results of the proportion achieving 10% weight loss in included trials.


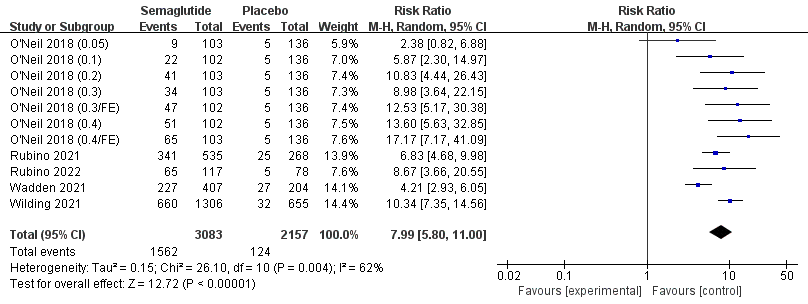


Figure S4. Meta-analysis results of the proportion achieving 15% weight loss in included trials.


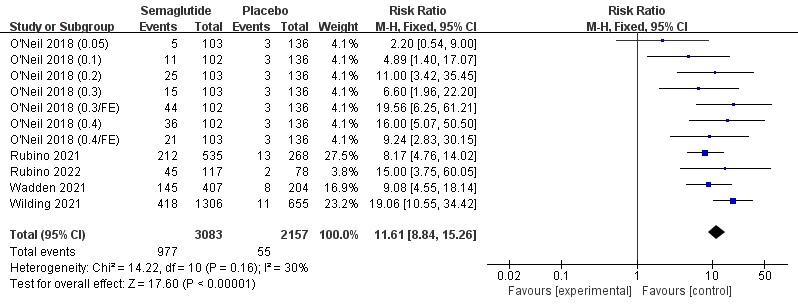


Figure S5. Meta-analysis results of the proportion achieving 20% weight loss in included trials.


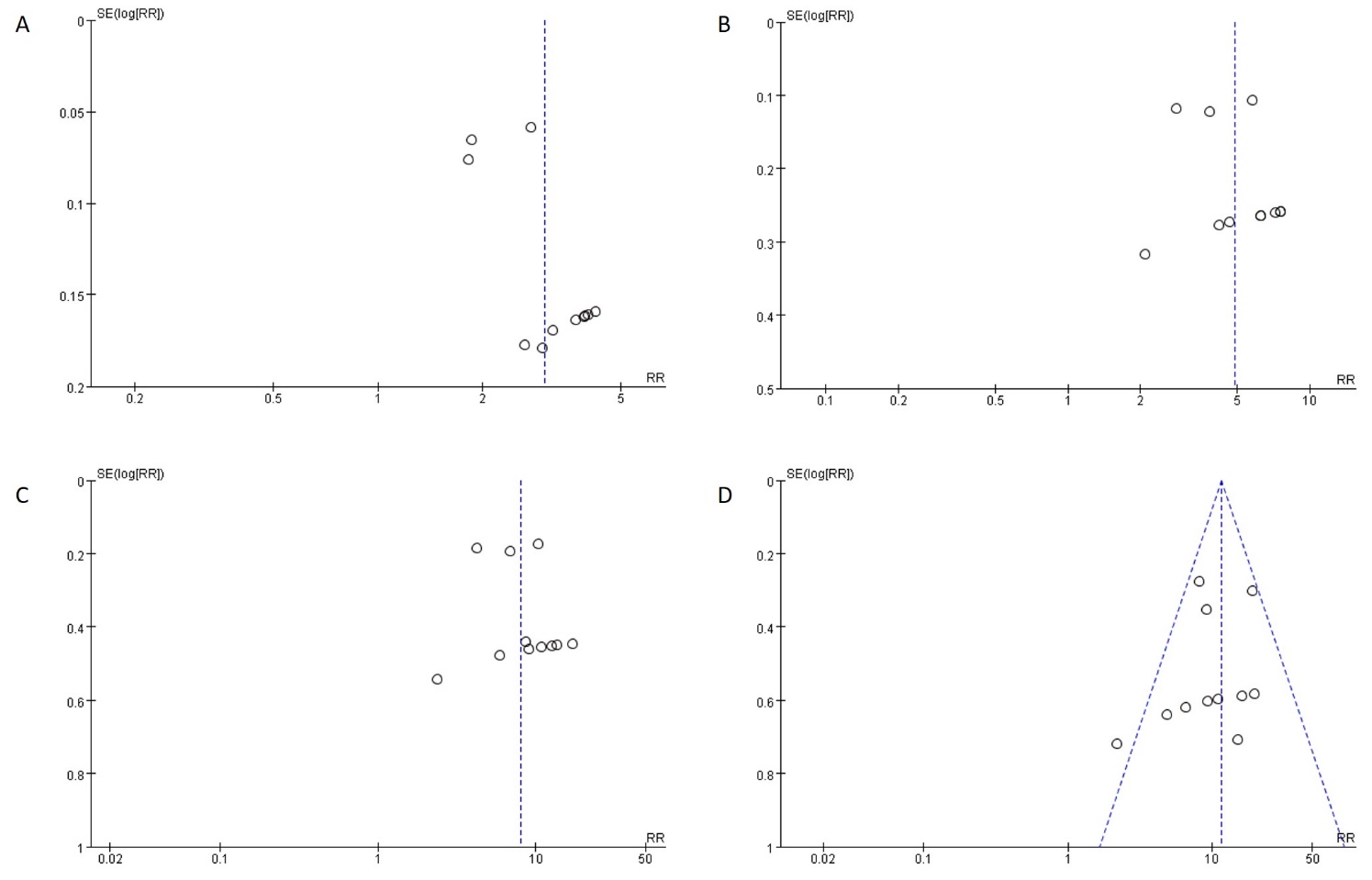


Figure S6. Funnel plots of the proportion achieving 5% (A), 10% (B), 15% (C) and 20% (D) weight loss in included trials.


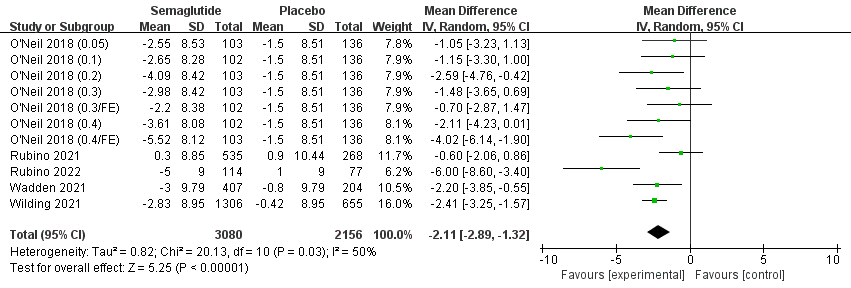


Figure S7. Meta-analysis results of DBP change (mm Hg) in included trials.


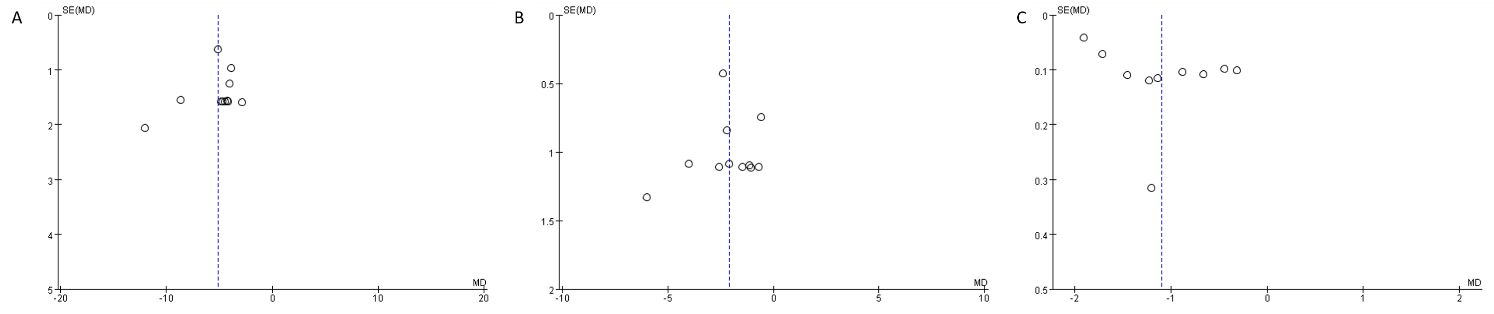


Figure S8. Funnel plots of SBP (A) (mm Hg), DBP (B) (mm Hg) and CRP (C) (mg/L) changes in included trials.


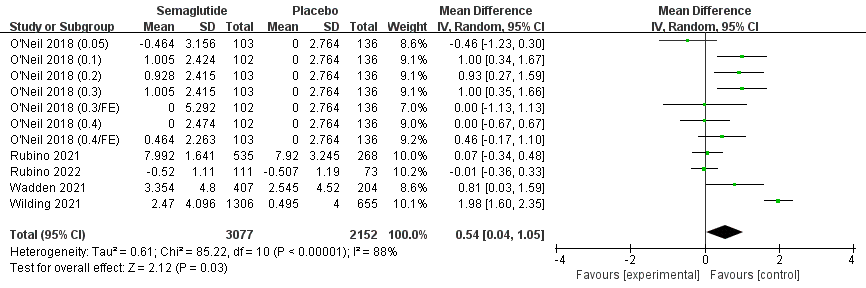


Figure S9. Meta-analysis results of HDL change (mg/dl) in included trials.


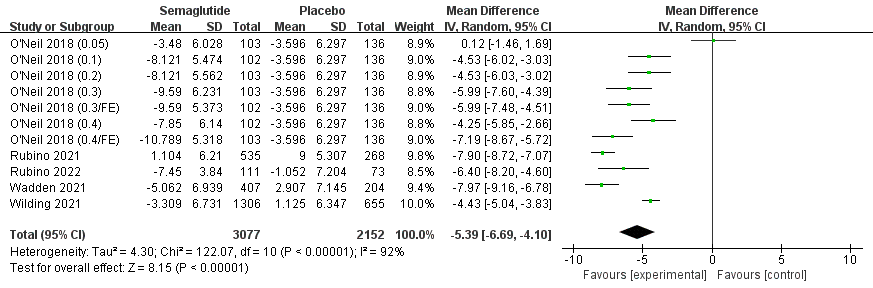


Figure S10. Meta-analysis results of LDL change (mg/dl) in included trials.


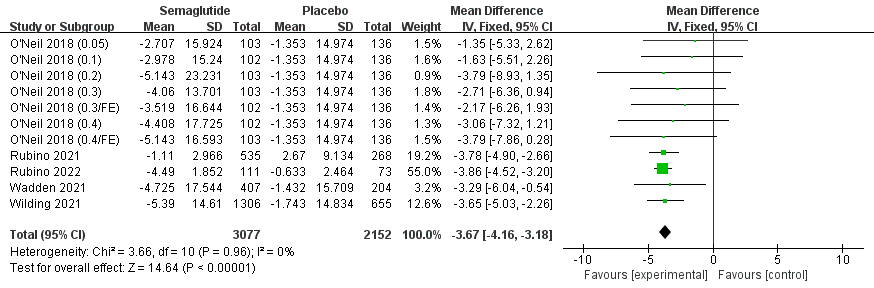


Figure S11. Meta-analysis results of VLDL change (mg/dl) in included trials.


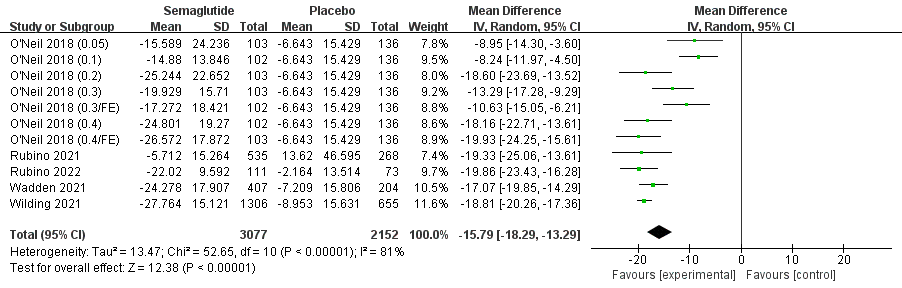


Figure S12. Meta-analysis results of TG change (mg/dl) in included trials.


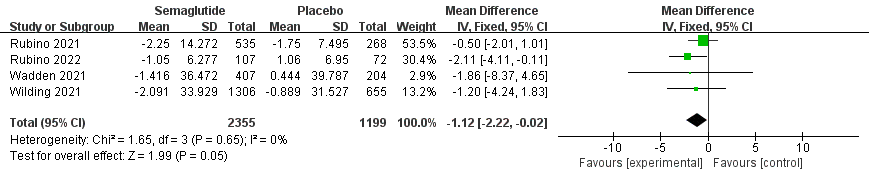


Figure S13. Meta-analysis results of FFA change (mg/dl) in included trials.


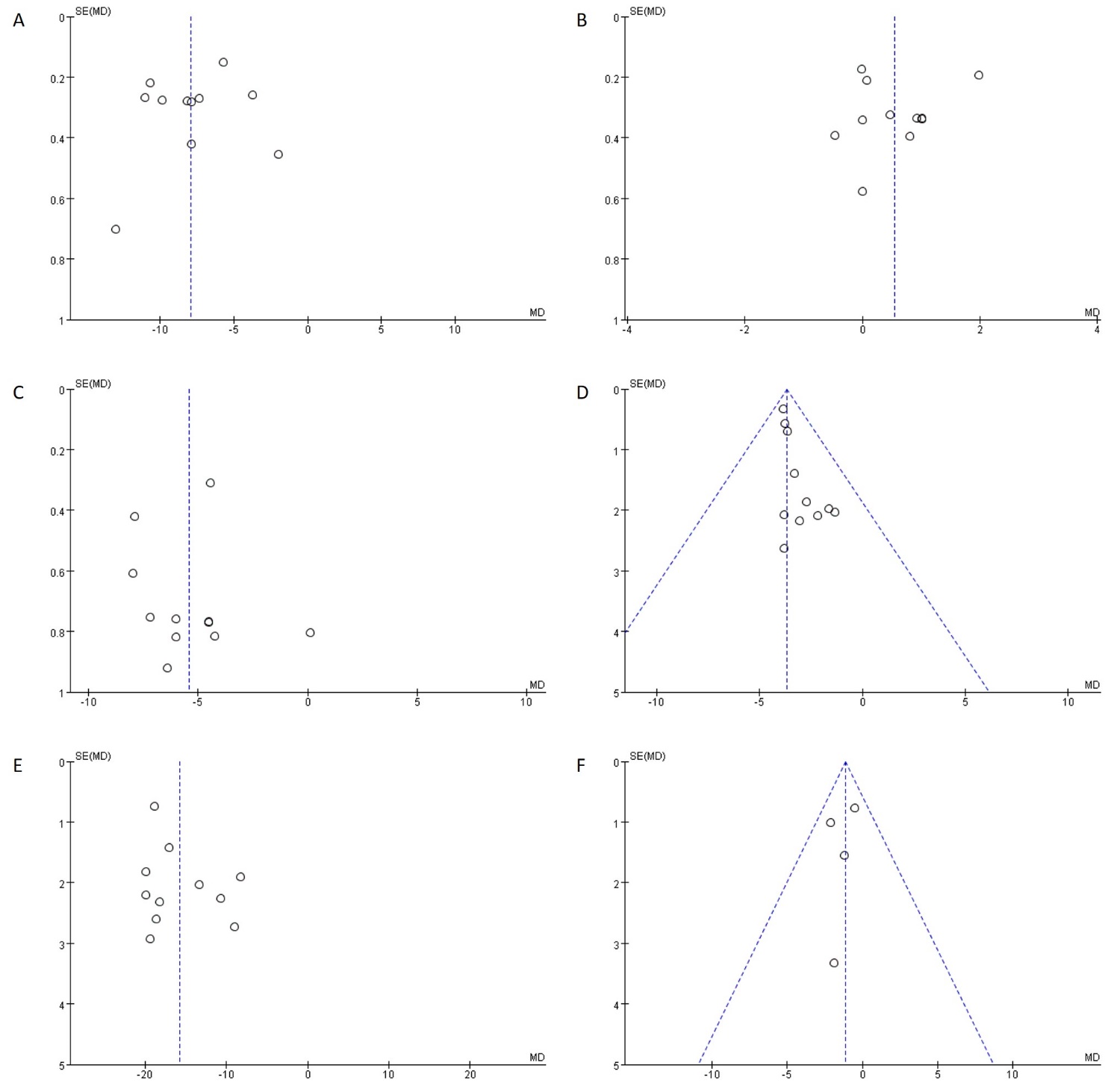


Figure S14. Funnel plots of TC (A), HDL (B), LDL (C), VLDL (D) TG (E) and FFA (F) changes (mg/dl) in included trials.


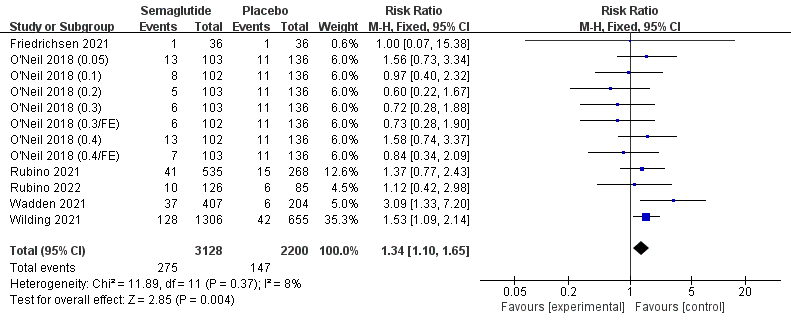


Figure S15. Meta-analysis results of the proportion with SAEs in included trials.


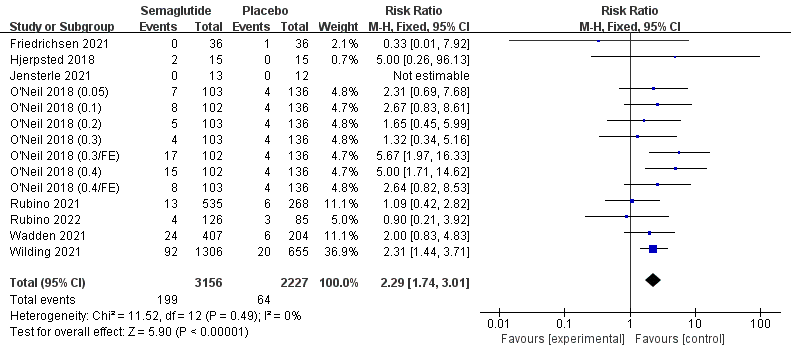


Figure S16. Meta-analysis results of the proportion with DAEs in included trials.


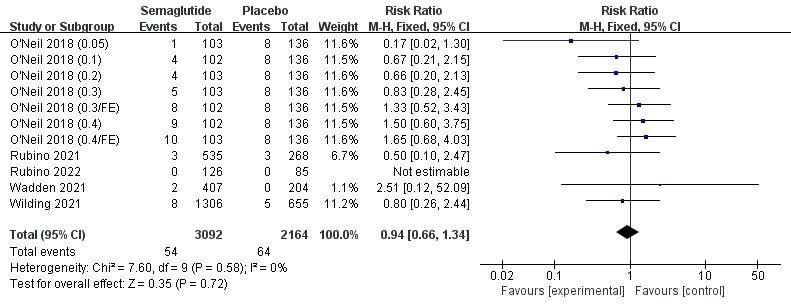


Figure S17. Meta-analysis results of the proportion with hypoglycemia between the semaglutide and placebo in included trials.


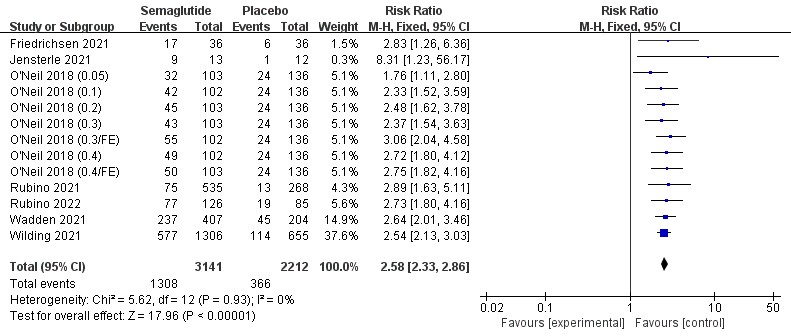


Figure S18. Meta-analysis results of the proportion with nausea between the semaglutide and placebo in included trials.


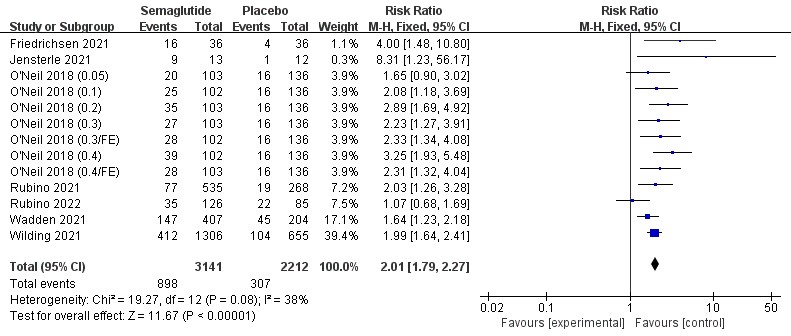


Figure S19. Meta-analysis results of the proportion with diarrhea between the semaglutide and placebo in included trials.


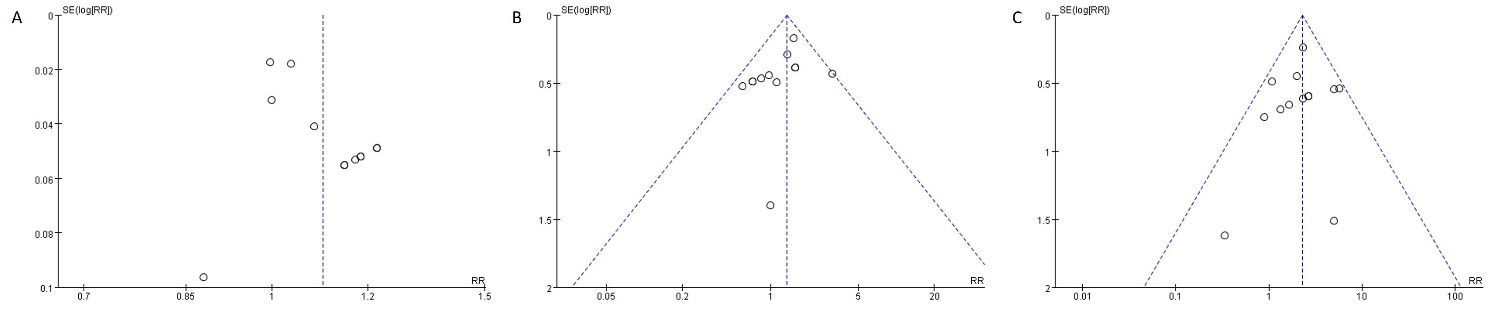


Figure S20. Funnel plots of the proportion with AEs (A), SAEs (B) and DAEs (C) in included trials.


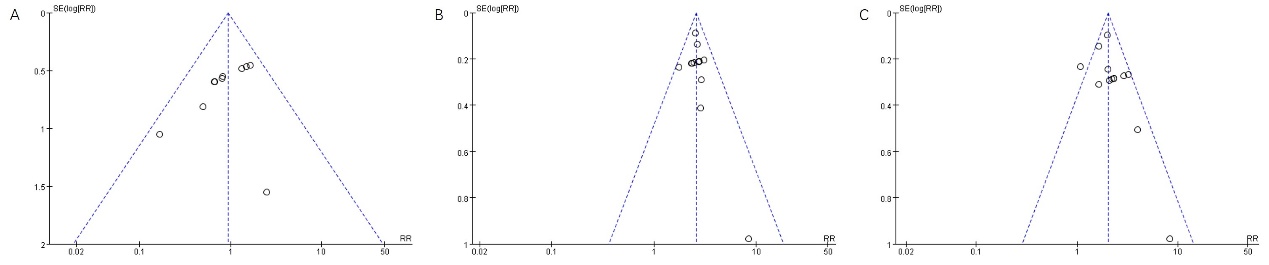


Figure S21. Funnel plots of the proportion with hypoglycemia (A), nausea (B) and diarrhea (C) in included trials.

Figure S22. Sensitivity analysis of RBW change (%) in included trials.

Figure S23. Sensitivity analysis of the proportion achieving 5% weight loss in included trials.
